# Treatment patterns and drug survival for generalized pustular psoriasis: A patient journey study using a Japanese claims database

**DOI:** 10.1111/1346-8138.17097

**Published:** 2024-01-12

**Authors:** Yayoi Tada, Jia Guan, Ryoko Iwasaki, Akimichi Morita

**Affiliations:** ^1^ Department of Dermatology Teikyo University Hospital Tokyo Japan; ^2^ Boehringer Ingelheim Pharmaceuticals Inc. Ridgefield Connecticut USA; ^3^ Nippon Boehringer Ingelheim Co., Ltd. Tokyo Japan; ^4^ Department of Geriatric and Environmental Dermatology Nagoya City University Graduate School of Medical Sciences Nagoya Japan

**Keywords:** generalized pustular psoriasis, Japan, rare diseases, skin diseases, therapeutics

## Abstract

Generalized pustular psoriasis (GPP) is a potentially life‐threatening skin disease. Although several medications are approved for treating GPP in Japan, there are limited data on real‐world treatment patterns or drug survival (the number of prescribed days of treatment). This retrospective cohort study describes drug survival and treatment patterns of patients with newly diagnosed GPP (International Classification of Diseases, 10th Revision code L40.1), and ≥1 year of follow‐up, using de‐identified claims data (Medical Data Vision Co., Ltd.) from January 2016 to August 2021. Most (97.0%) of the 434 Japanese patients received first‐line therapy of etretinate (26.4%), topical medications (14.7%), or cyclosporin (14.3%); 80.0% and 60.1% of patients received a second and third line of therapy (LOT), respectively. Use of etretinate (12.6%) and cyclosporin (5.9%) decreased in second‐line therapies, whereas use of biologics (interleukin [IL]‐17, 14.3%; IL‐23 inhibitors, 7.6%) and topical medications (22.1%) increased or remained consistent. Approximately 50% of biologics were prescribed in combination with systemic medications or systemic corticosteroids. Median (range) time to next therapy (TTNT) was 2.8 (0.03–48.07) months for first‐line therapy and 3.3 (0.03–52.97) months for all other LOTs. TTNT was longer for combination therapies (up to 16.5 months) compared with monotherapies (up to 7.5 months). Biologics exhibited longer drug survival with fewer treatment episodes compared with non‐biologic systemic medications. Among frequently used therapies, the median (95% confidence interval) drug survival was 8.8 (5.8–11.8) months for etretinate, 4.3 (2.2–6.9) months for systemic corticosteroids, and 19.6 (16.1–26.7) months for secukinumab. Treatment patterns varied considerably, highlighting the need for treatment algorithms and effective, well‐tolerated medications to support patients to help them remain on long‐term therapy.

## 1 INTRODUCTION

Generalized pustular psoriasis (GPP) is a rare, potentially life‐threatening skin disease characterized by episodes of widespread eruption of sterile pustules, occurring with or without systemic inflammation.^1^, ^2^, ^3^, ^4^ The clinical course of GPP is unpredictable, presenting as recurrent flares or a persistent disease with intermittent flares, and a broad spectrum of symptom severity between patients and individual flares.^2^, ^4^ Therefore, some patients require a long‐term treatment plan.

Although several medications are approved for GPP in Japan, the evidence for these approvals is largely derived from small, open‐label clinical trials and case reports.^2^, ^5^, ^6^, ^7^, ^8^ Given that only a few treatments have been evaluated in randomized controlled trials, no strong evidence is available to establish treatment algorithms or guidance on the optimal order of optimal treatment; consequently, GPP treatment varies considerably.^2^, ^4^, ^9^ Available management guidelines and recommendations generally suggest cyclosporin, retinoids, infliximab, or methotrexate as first‐line therapies.^2^, ^9^

For diseases requiring long‐term treatment, prolonged drug survival (the length of time a patient remains on a specific drug) can provide insight into treatment effectiveness and safety.^10^ Although some reports indicate that patients with GPP are more likely to be treated with combination therapy compared with psoriasis vulgaris (PsV),^11^, ^12^ detailed analysis of GPP treatment combinations and treatment patterns by line of therapy (LOT) in clinical practice in Japan has not been conducted.^2^ Similarly, although GPP is considered a poor prognostic factor for drug survival in treating PsV, drug survival data for GPP‐specific treatment options are needed.^10^, ^13^

This study was conducted to explore treatment patterns and drug survival of GPP treatments in Japan to provide further insight into the LOT for GPP and biologic treatment switching.

## 2 METHODS

### 2.1 Study design

A retrospective cohort study was conducted using a large‐scale hospital‐based database (Medical Data Vision Co., Ltd, Tokyo, Japan). The database covers approximately 20% of hospitals using the Diagnosis Procedure Combination (DPC) system/Per‐Diem Payment System in Japan, comprising >30 million people as of December 2020. De‐identified data include patient demographics, diagnosis, laboratory tests, procedures, prescriptions, admissions, and discharge information. The study protocol was approved by the ethics committee of AMC Nishi‐Umeda Clinic (Osaka, Japan; AMC‐BI‐21‐010).

### 2.2 Patients

Patients newly diagnosed with GPP (International Classification of Diseases, 10th Revision code L40.1) between January 1, 2016, and August 31, 2021, were eligible for the study. Patients were excluded if they only had one confirmed GPP diagnosis, could not be followed for >1 year after diagnosis (except those who died within 1 year), or had unclear or no information on GPP diagnosis history.

The date of initial GPP diagnosis was defined as the index date; the baseline period was a year before the index date. Patients were followed up until the end of the study (August 31, 2021), death, or loss to follow‐up, whichever came first. Full details are provided in Methods S1.

### 2.3 Outcomes and measurements

Demographic characteristics were recorded at the index date, or most recent record before the index date, during the baseline period. The Charlson Comorbidity Index (CCI) scores for comorbidity evaluation were calculated based on the Quan method.^14^

As there are no established algorithms to identify the LOT for GPP treatment, we created and optimized a complex algorithm to capture treatment patterns in the real‐world setting, based on simulation results and expert opinions. All medications for GPP were classified into main or supplementary medications. Medication switch, add‐on, and discontinuation were considered for monotherapy and combination therapy.^15^ Supplementary medications in combination therapy did not change the LOT; supplementary medications remained as one LOT in monotherapy. Due to the number of specific regimens, these were categorized into general regimens according to medication category and defined prioritization (Table 1). Prioritization order was decided according to which drugs were considered the main drugs in combination therapy.

**TABLE 1:** Prioritization of treatment.

Treatment category 1	Treatment category 2	Prioritization order
Biologics	IL‐17 inhibitors	1
	TNF‐α inhibitors	2
	IL‐23 inhibitors^a^	3
Systemic oral medications	Etretinate	4
	Cyclosporin	5
	Corticosteroids	7
	Apremilast^a^	8
	Methotrexate	9
	Other oral medications^b^	11
Apheresis/plasma exchange	Apheresis/plasma exchange	6
Topical therapy^b^	Topical therapy	10
Arthritis treatment^b^	Arthritis treatment	12

Abbreviations: GPP, generalized pustular psoriasis; IL, interleukin; TNF‐α, tumor necrosis factor alpha.

^a^ The IL‐23 inhibitor, ustekinumab and apremilast are not approved for GPP in Japan but are used for patients with GPP who also have psoriasis vulgaris.

^b^ Topical medications, other oral medication, and arthritis treatment were defined as supplementary drugs.

Time to next therapy (TTNT) and drug survival analyses were performed as proxies for clinical outcomes. TTNT was defined as the start date of one LOT to the start date of the subsequent LOT. Drug survival analysis investigated patient adherence to a specific medication. We defined drug discontinuation as a 3‐month interval for biologics and a 2‐month interval for oral drugs. Frequency of switching to other biologics was measured according to the last episode of each biologic. A full list of treatments and study definitions are provided in Tables S1 and S2, respectively.

### 2.4 Statistical analysis

Descriptive statistics were calculated for baseline characteristics and treatment patterns. TTNT and drug survival analyses were performed using the Kaplan–Meier method. For TTNT, the last LOT for each patient was defined as “censor,” whereas other LOTs were defined as “event.” For drug survival, when multiple episodes of one medication were recorded by one patient, the end date of the last episode was defined as “censor,” whereas other episodes were defined as “event.” Drug survival of all episodes was estimated among recipients of that medication. All statistical analyses were performed using SAS version 9.4 (SAS Institute Inc., Cary, NC, USA).

## 3 RESULTS

### 3.1 Patients

In total, 434 Japanese patients with GPP were included in the analyses (Table 2; Table S3). Patients had a mean (standard deviation) age of 57.2 (19.9) years, and 47.5% were male. Most patients (61.8%) attended large hospitals (≥500 beds) and had a CCI score of 0–1 (69.8%). PsV was the most frequent comorbidity (36.6%), and lidocaine hydrochloride/adrenaline was the most frequent concomitant medication (37.8%).

**TABLE 2:** Demographics and baseline characteristics of patients newly diagnosed with GPP between January 1, 2016, and August 31, 2021.

Demographics and baseline characteristics	Patients (*N* = 434)
Age, mean (SD), years	57.2 (19.9)
Total follow‐up, median (IQR), days	809 (570–1206)
Charlson Comorbidity Index score, *n* (%)	
0	211 (48.6)
1	92 (21.2)
2	63 (14.5)
3	25 (5.8)
≥4	43 (9.9)
Most common comorbidities,^a^ *n* (%)	
Psoriasis vulgaris	159 (36.6)
Hypertension	79 (18.2)
Low back pain	76 (17.5)
Most common concomitant medications, *n* (%)	
Lidocaine hydrochloride/adrenaline	164 (37.8)
Isotonic sodium chloride solution	140 (32.3)
Acetaminophen	110 (25.4)

Abbreviations: GPP, generalized pustular psoriasis; ICD‐10, International Classification of Diseases, 10th Revision; IQR, interquartile range; SD, standard deviation.

^a^ Identified by disease codes as follows: ICD‐10 code L40.0 psoriasis vulgaris 6961004; standard disease code 8833421 hypertension; 8840829 low back pain; 8840042 constipation; 6923002 eczema. Standard disease code 6961004 psoriasis vulgaris was *n* = 128 (29.5%).

### 3.2 Treatment patterns

**FIGURE 1:**
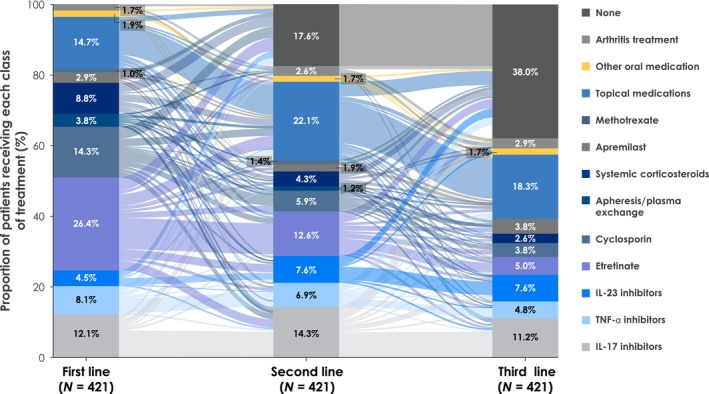
Sankey plot of the treatment pathways of the first three lines of therapy in patients with newly diagnosed GPP (cohort number = 421). Each line represents the treatment pathway at each line of therapy. GPP, generalized pustular psoriasis; IL, interleukin; TNF‐α, tumor necrosis factor alpha.

The Sankey plot shows treatment patterns for general regimens (Figure 1). Of the 434 patients, 421 (97.0%) received first‐line therapy; 80.0% and 60.1% of patients received a second and third LOT, respectively. The most common first‐line therapies were oral medications, including etretinate (26.4%) and cyclosporin (14.3%), topical medications (14.7%), and interleukin (IL)‐17 inhibitors (12.1%; Figures S1 and S2a,b). In subsequent therapies (e.g., second line), use of oral medications, including etretinate (12.6%) and cyclosporin (5.9%), decreased, whereas use of topical medications (22.1%) and biologic‐based therapies, including IL‐17 inhibitors (14.3%), tumor necrosis factor alpha (TNF‐α) inhibitors (6.9%), and IL‐23 inhibitors (7.6%), increased or remained consistent. Among the biologics, the most common treatments in each category were secukinumab (IL‐17 inhibitor), adalimumab (TNF‐α inhibitor), and guselkumab (IL‐23 inhibitor; Figure S2b). Among patients who received first‐line etretinate‐based therapies, further etretinate, or a switch to topical‐, biologic‐, or cyclosporin‐based therapies were common as second LOT.

### 3.3 Treatment combinations

**FIGURE 2:**
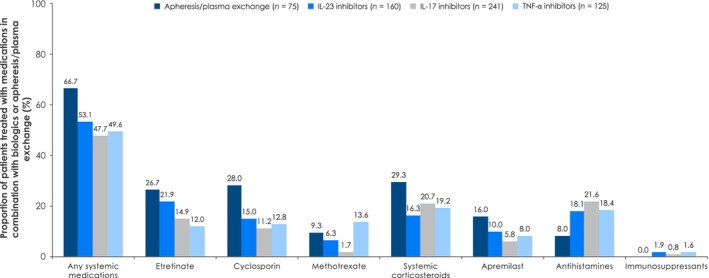
Combination patterns of GPP treatments: Combinations of biologics or apheresis/plasma exchange with other systemic medications. GPP, generalized pustular psoriasis; IL, interleukin; TNF‐α, tumor necrosis factor alpha.

Combination patterns of biologics or apheresis/plasma exchange were common (Figure 2). Approximately 50% of biologics were prescribed in combination with either systemic medications or systemic corticosteroids. IL‐17 and TNFα inhibitors were frequently combined with antihistamines (21.6% and 18.4%, respectively) or systemic corticosteroids (20.7% and 19.2%, respectively). IL‐23 inhibitors were frequently combined with etretinate (21.9%) or antihistamines (18.1%). Approximately 30% of systemic medications were combined with topical medications, most frequently with topical corticosteroids (17.3–32.4%) or vitamin D3 (alone or in combination with steroid medication, e.g., betamethasone, 4.0–22.1%; Figure S3a). Combinations of methotrexate with non‐steroidal anti‐inflammatory drugs were also common (32.9%; Figure S3b).

### 3.4 Time to next therapy

Median (range) TTNT was 2.8 (0.03–48.07) months for first‐line therapy and 3.3 (0.03–52.97) months for all LOTs (Table S4). Across all LOTs, most GPP treatments were used in combination, typically a general regimen (e.g., etretinate) plus supplementary drugs (e.g., tocilizumab); the resulting combination therapies had a longer TTNT than monotherapies (Table 3). Among the most frequent specific regimens, across all lines, median TTNTs were shorter for etretinate (2.1 months) and cyclosporin (2.7 months) alone, but longer for etretinate plus a supplementary drug (8.1 months) and cyclosporin plus a supplementary drug (9.9 months). Similarly, median TTNT for IL‐17 inhibitors was 5.7 months for monotherapy and 11.4 months for IL‐17 inhibitor plus a supplementary drug. In addition, systemic corticosteroids were frequently used as monotherapy, but had a short median TTNT.

**TABLE 3:** Time to next therapy of frequently used specific regimens across all lines.

General regimen	Specific regimen^a^	Time to next therapy, median (range), months
Apheresis/plasma exchange	Apheresis/plasma exchange (*n =* 12)	2.0 (1.2–8.5)
	Apheresis/plasma exchange + cyclosporin (*n =* 5)	2.5 (1.0–3.5)
Topical medications	Topical medications + supplementary drug (*n =* 184)	2.3 (0.03–37.0)
	Topical medications (*n =* 158)	4.1 (0.5–30.7)
Etretinate	Etretinate + supplementary drug (*n =* 41)	8.1 (3.3–48.1)
	Etretinate (*n =* 27)	2.1 (0.1–27.4)
	Etretinate + systemic corticosteroids (*n =* 19)	1.3 (0.03–10.4)
	Cyclosporin + etretinate (*n =* 15)	0.97 (0.03–2.2)
	Etretinate + systemic corticosteroids + supplementary drug (*n =* 12)	6.6 (3.1–30.9)
	Cyclosporin + etretinate + supplementary drug (*n =* 9)	6.2 (3.1–53.0)
	Apremilast + etretinate (*n =* 8)	1.4 (0.3–3.1)
	Cyclosporin + etretinate + systemic corticosteroids (*n =* 6)	1.8 (1.1–2.8)
Cyclosporin	Cyclosporin (*n =* 31)	2.7 (0.7–24.8)
	Cyclosporin + supplementary drug (*n =* 18)	9.9 (3.5–37.0)
	Cyclosporin + systemic corticosteroids (*n =* 11)	2.1 (0.1–15.4)
	Cyclosporin + systemic corticosteroids + supplementary drug (*n =* 8)	15.4 (3.2–25.4)
	Apremilast + cyclosporin (*n =* 6)	1.6 (0.5–2.9)
Methotrexate	Methotrexate + supplementary drug (*n =* 8)	7.1 (4.7–14.0)
	Methotrexate (*n =* 7)	7.5 (2.1–19.7)
Systemic corticosteroids	Systemic corticosteroids (*n =* 36)	2.1 (0.03–20.6)
	Systemic corticosteroids + supplementary drug (*n =* 13)	7.3 (3.1–24.5)
Apremilast	Apremilast (*n =* 15)	1.9 (1.2–9.8)
	Apremilast + supplementary drug (*n =* 13)	16.5 (4.2–25.4)
IL‐17 inhibitors	IL‐17 inhibitors + supplementary drug (*n =* 37)	11.4 (3.3–46.1)
	IL‐17 inhibitors (*n =* 27)	5.7 (1.3–40.6)
	IL‐17 inhibitors + systemic corticosteroids (*n =* 13)	1.4 (0.1–3.4)
	IL‐17 inhibitors + systemic corticosteroids + supplementary drug (*n =* 13)	5.1 (3.3–46.7)
	Cyclosporin + IL‐17 inhibitors (*n =* 14)	1.8 (0.1–5.4)
	Etretinate + IL‐17 inhibitors (*n =* 9)	1.4 (0.03–2.7)
	Etretinate + IL‐17 inhibitors + supplementary drug (*n =* 6)	11.7 (3.3–15.2)
	IL‐12/IL‐23 inhibitors + IL‐17 inhibitors (*n =* 6)	2.5 (1.2–12.4)
IL‐23 inhibitors	IL‐23 inhibitors (*n =* 10)	4.0 (1.4–17.6)
	IL‐23 inhibitors + supplementary drug (*n =* 10)	8.3 (3.6–15.5)
	Etretinate + IL‐23 inhibitors (*n =* 8)	2.1 (0.03–9.4)
	Etretinate + IL‐23 inhibitors + supplementary drug (*n =* 5)	13.4 (4.4–19.3)
	Cyclosporin + IL‐23 inhibitors (*n =* 5)	3.3 (0.5–9.4)
TNF‐α inhibitors	TNF‐α inhibitors (*n =* 17)	4.2 (1.0–16.1)
	TNF‐α inhibitors + supplementary drug (*n =* 16)	10.4 (3.7–28.8)
	Systemic corticosteroids + TNF‐α inhibitors (*n =* 8)	2.3 (1.1–5.3)
	Etretinate + TNF‐α inhibitors (*n =* 5)	1.7 (0.03–3.2)
Arthritis treatment	Arthritis treatment (*n =* 33)	5.8 (0.5–34.3)
Other oral medications	Other oral medications (*n =* 28)	2.9 (0.2–31.0)
	Other oral medications + supplementary drug (*n =* 6)	2.2 (0.5–35.5)

Abbreviations: IL, interleukin; TNF‐α, tumor necrosis factor alpha.

^a^ Supplementary drugs are topical medications, other oral medications, and arthritis treatments.

### 3.5 Drug survival

Generally, biologics exhibited longer drug survival with fewer treatment episodes compared with non‐biologic systemic medications (Table 4). Median drug survival for frequently prescribed non‐biologic systemic medications (e.g., systemic corticosteroids [4.3 months] and etretinate [8.8 months]) was shorter than for common biologics (e.g., secukinumab [19.6 months] and guselkumab [14.0 months]). In addition, >65% of patients treated with infliximab, ixekizumab, secukinumab, or ustekinumab were treated for >1 year, and < 40% of patients continued treatment for >2 years. Conversely, the proportion of patients treated with non‐biologic systemic medications for >1 year ranged from 24.6% to 45.5%.

**TABLE 4:** Drug survival of systemic medications and biologics.

Drug	Number of patients^a^	Number of treatment episodes	Median drug survival (95% CI), months	Patients with treatment duration	
				>1 year, *n* (%)	>2 years, *n* (%)
Systemic medication (non‐biologics)					
Apremilast	77	87	10.1 (3.9–15.0)	35 (45.5)	15 (19.5)
Cyclosporin	116	139	5.6 (4.0–7.9)	39 (33.6)	24 (20.7)
Etretinate	147	199	8.8 (5.8–11.8)	58 (39.5)	27 (18.4)
Methotrexate	34	91	12.0 (4.9–19.3)	15 (44.1)	8 (23.5)
Systemic corticosteroid	156	192	4.3 (2.2–6.9)	49 (31.4)	30 (19.2)
Antihistamines	285	426	8.2 (5.6–10.1)	109 (38.2)	48 (16.8)
Immunosuppressants^b^	65	79	0.8 (0.5–3.7)	16 (24.6)	4 (6.2)
TNF‐α inhibitors					
Adalimumab	33	47	17.8 (6.5–22.4)	19 (57.6)	10 (30.3)
Certolizumab pegol	6	6	8.4 (4.7–12.1)	2 (33.3)	0
Infliximab	23	23	22.1 (9.3–26.1)	15 (65.2)	9 (39.1)
IL‐17 inhibitors					
Brodalumab	24	34	12.6 (8.2–20.8)	13 (54.2)	8 (33.3)
Ixekizumab	33	45	15.5 (10.7–23.8)	22 (66.7)	11 (33.3)
Secukinumab	58	66	19.6 (16.1–26.7)	43 (74.1)	23 (39.7)
IL‐23 inhibitors					
Guselkumab	49	55	14.0 (9.3–19.8)	27 (55.1)	14 (28.6)
Risankizumab	26	26	13.5 (10.1–15.6)	14 (53.8)	1 (3.8)
Ustekinumab	14	14	16.8 (11.2–19.6)	10 (71.4)	3 (21.4)

Abbreviations: CI, confidence interval; IL, interleukin; TNF‐α, tumor necrosis factor alpha.

^a^ *N* represents the total number of patients receiving each drug.

^b^ Immunosuppressants are tacrolimus, dapsone, minocycline, or azathioprine.

### 3.6 Bio‐switching

**FIGURE 3:**
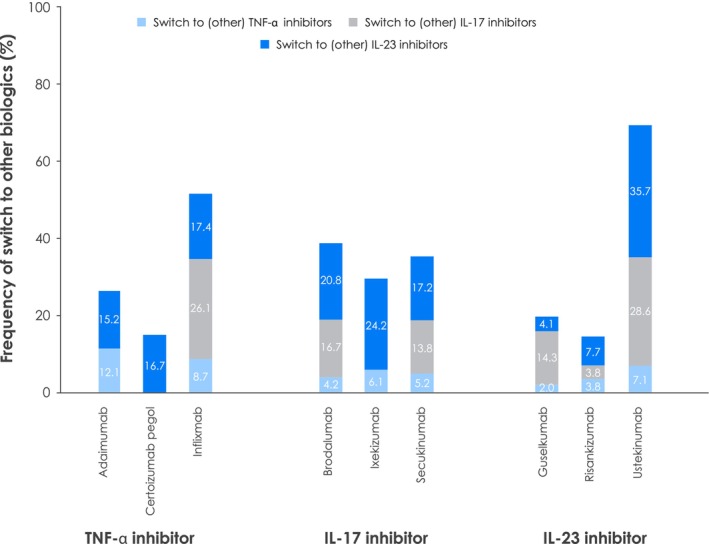
Patterns in switching GPP treatment from one biologic to another. GPP, generalized pustular psoriasis; IL, interleukin; TNF‐α, tumor necrosis factor alpha.

Treatment switching occurred in every biologic class, often to other biologics, and was most common among patients treated with infliximab (52.2%) or ustekinumab (71.4%) (Figure 3). Over 15% of patients treated with TNF‐α inhibitors (15.2–17.4%) and > 17% treated with IL‐17 inhibitors (17.2–24.2%) were switched to IL‐23 inhibitors; few patients switched to other TNF‐α inhibitors (≤12.1%).

### 3.7 Subgroup analysis: Patients with versus without PsV

**FIGURE 4:**
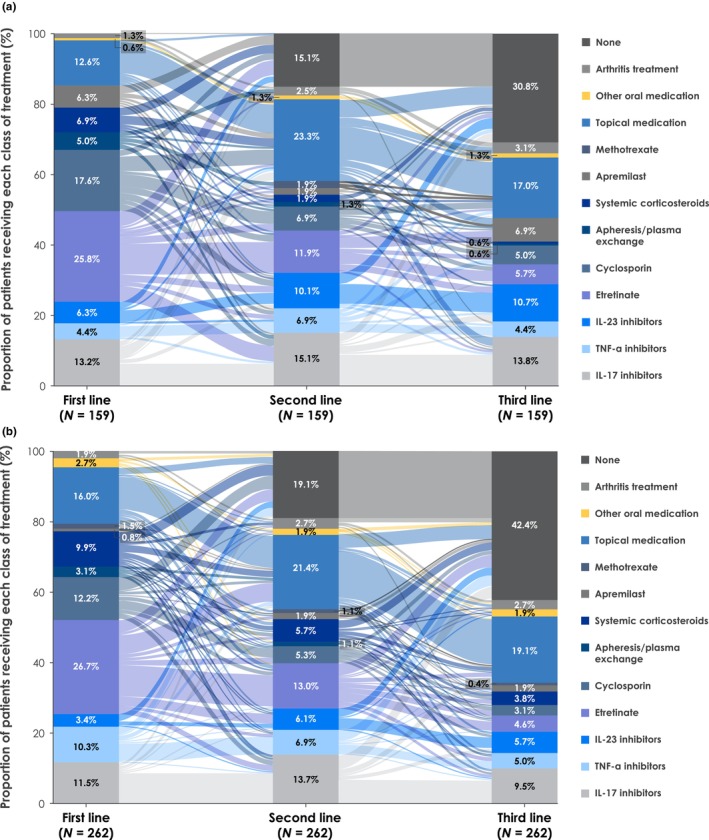
Treatment pathway in patients with newly diagnosed GPP (a) with PsV and (b) without PsV. GPP, generalized pustular psoriasis; IL, interleukin; PsV, psoriasis vulgaris; TNF‐α, tumor necrosis factor alpha.

This subgroup analysis included 159 (36.6%) patients with GPP with PsV and 275 (63.4%) patients with GPP without PsV. More patients with GPP with PsV were treated with IL‐17 inhibitors, IL‐23 inhibitors, cyclosporin, and apremilast‐based therapies than those without PsV across all LOT. Conversely, more patients without PsV were treated with TNF‐α inhibitors and systemic corticosteroid‐based therapies (Figure 4; Figures S4 and S5). Median drug survival was generally longer for patients without versus with PsV for both biologics (e.g., brodalumab: without PsV 25.8 months; with PsV, 11.4 months) and non‐biologics (e.g., apremilast: without PsV, 10.1 months; with PsV, 8.6 months), and more patients without PsV achieved drug survival of >12 months (Table S5). Switching between biologics was more frequent in patients with GPP with PsV than in those with GPP without PsV. Ustekinumab had the highest proportion of patients switch in both cohorts (with PsV, 100.0%; without PsV, 50.0%), followed by infliximab (with PsV, 75.0%; without PsV, 47.4%). The highest proportion of patients with GPP with PsV switched to IL‐23 inhibitors, and the highest proportion of patients with GPP without PsV switched to IL‐17 inhibitors (Figure S6).

### 3.8 Subgroup analysis: Elderly patients

**FIGURE 5:**
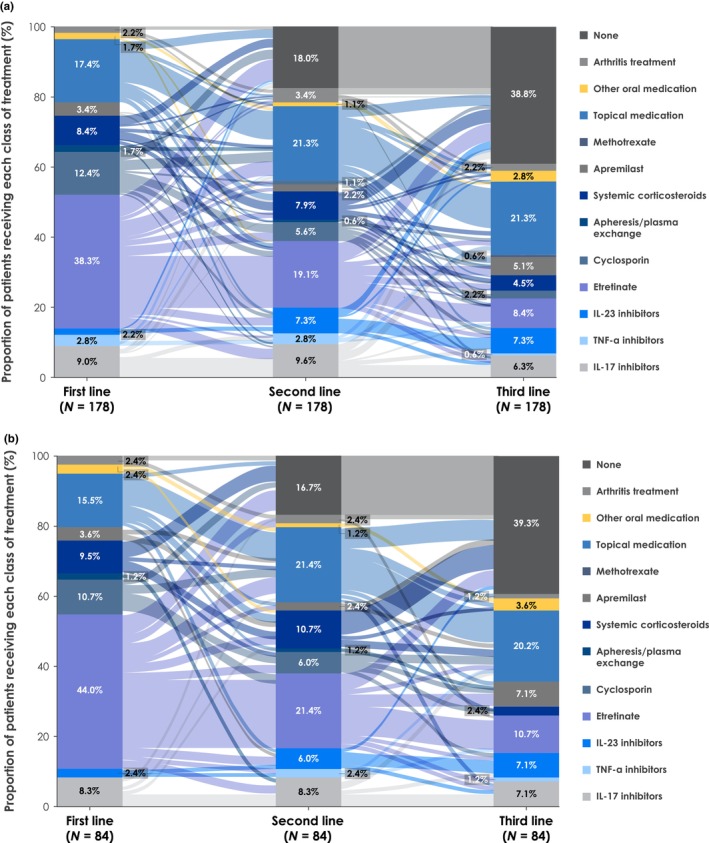
Treatment pathway in patients with newly diagnosed GPP aged (a) ≥65 years and (b) ≥75 years. GPP, generalized pustular psoriasis; IL, interleukin; TNF‐α, tumor necrosis factor alpha.

This subgroup analysis included 185 (42.6%) patients aged ≥65 years and 87 (20.0%) patients aged ≥75 years. Use of biologic‐based therapies as first‐line therapy was less common among elderly patients than in the overall population (Figure 5; Figure S7). Notably, TNF‐α inhibitor‐based regimens were not used as first‐line therapy in the ≥75‐year‐old cohort (Figure S7b). Use of biologics increased from second‐line therapy onwards, but it remained lower than in the overall population; the exception was IL‐23 inhibitors, which were more commonly prescribed for elderly patients than in the overall population (Figure 5; Figures S7 and S8). The proportion of elderly patients treated with biologics, with a median drug survival of >1 year, was similar to the overall population (Table S6), suggesting that biologics were well tolerated by elderly patients with GPP. For patients aged ≥65 years with GPP, ustekinumab had the highest switch frequency (75.0%), whereas secukinumab was the most frequently switched biologic in patients aged ≥75 years (66.6%). Bio‐switching was relatively common among both elderly cohorts (Figure S9a,b).

## 4 DISCUSSION

Our analysis of GPP treatment patterns in Japan identified that, in accordance with the Japanese Dermatological Association (JDA) treatment algorithm, etretinate‐ and cyclosporin‐based therapies were common first‐line therapies. This indicates that conventional therapy is still widely used by physicians,^2^ while use of biologic‐based therapies (including secukinumab, adalimumab, and guselkumab) are increasing in subsequent LOTs. Although biologics are recommended for patients not responding to conventional therapy,^2^ current JDA guidelines largely reflect treatment options available as of August 2013.^5^, ^16^, ^17^, ^18^, ^19^, ^20^, ^21^ This lack of up‐to‐date guidance was illustrated by the diverse treatment sequencing, with no discernible treatment algorithm, and treatment selection dependent on the physician. Switching to biologics may reflect a need to improve patients' symptoms through selective suppression of GPP‐associated inflammatory pathways, using targeted treatments.^22^

Lidocaine hydrochloride/adrenaline (a local anesthetic) was the most frequent concomitant medication, probably because it is used during biopsies that are performed for diagnostic testing.^23^, ^24^ Combination therapies, including supplementary drugs, generally had a longer TTNT than monotherapies, indicating that current GPP monotherapies are not as efficacious in managing GPP in the long term. Approximately 30% of systemic medications were combined with topical medications, whereas approximately 50% and 16–21% of biologics were combined with systemic medications and systemic corticosteroids, respectively. Dermatologists may consider biologic monotherapies to be inadequate, concluding that combinations with antihistamines, for example, are needed to control specific symptoms, such as itchiness. Further insight is required to understand which skin symptoms are the most challenging to treat.

Biologics generally had a longer drug survival with fewer treatment episodes than oral medications, which supports findings from Germany.^25^ Drug survival data of patients with psoriasis, including GPP, from two university hospitals in Japan (Fukuoka University and Jichi Medical University) have shown similar results to our study.^13^, ^26^, ^27^ Of these, Kishimoto et al. identified GPP as a negative predictor for biologic drug persistence,^13^ potentially due to the intermittent nature of GPP flares or treatment inefficacy. Based on our data, no treatment had a substantially long drug survival (0.8–22.1 months) reflecting the dynamic nature of GPP in which symptom resolution following treatment may reduce the likelihood of patients using the treatment as a long‐term therapy. Additionally, patients frequently switched biologics, which may be associated with the tendency of practitioners to switch from older biologics, such as infliximab and ustekinumab, to newly available biologics. Conversely, there are also some physicians who continue to use biologics as GPP maintenance therapy, in the same way as for treatment of PsV.

Treatment patterns varied according to the presence of PsV and age. Patients with GPP with PsV versus without PsV were more frequently treated with major drugs used in PsV, which may have been initiated before GPP diagnosis. However, it is also possible that practitioners recorded a diagnosis of PsV to facilitate treatment of GPP with medications approved for PsV but not approved to treat GPP, namely ustekinumab, and, therefore, provided a wider set of treatment options in this difficult‐to‐treat disease. Conversely, patients without PsV were more frequently treated with systemic corticosteroid and TNF‐α inhibitor‐based therapies, which are used to treat systemic symptoms.^2^ Differences in treatment pathways may be due to the variation in symptom severity, underlying disease mechanisms, or difficulty in controlling two distinct diseases. As elderly patients in Japan have a high rate of tuberculosis,^28^ decreased use of biologics in this cohort compared with the total study population likely reflects reluctance from physicians to prescribe biologics to patients in this age group, due to the increased risk of activating latent tuberculosis.^29^

There are several limitations to our analysis. Patient data were only captured if they were treated within the DPC hospital system. If a patient received care in another medical facility, this information was not captured. It is also possible that drug survival and treatment pattern data may be affected by the launch year of available therapies. Furthermore, we did not capture reasons for switching therapy, the effectiveness of the therapy, or flare occurrence in this study, as disease severity data are not recorded in the database. Disease severity is likely to have influenced treatment patterns and drug survival outcomes. However, the lack of disease severity data from the database meant that no conclusions could be drawn within the context of GPP disease severity. Missing information, miscoding, or underreporting of disorders could affect the study analysis; however, by focusing on patients treated with GPP‐related therapies, miscoding situations have most likely been excluded. Strengths of this study are that the data were derived from a large, deidentified claims database that covers over 30 million people. Patient treatment patterns and LOTs were determined using a novel algorithm, which considered switching, add‐on and discontinuation of treatment, and supplementary and main medications. This algorithm can be adapted and applied to other diseases in the future. Although data were available from 2011 onwards, we focused on data from 2016 to 2021 to reflect current treatment practices more accurately.

In conclusion, this analysis identified wide variations in treatment strategies, reflecting the complexity of managing GPP and the need to establish more detailed and up‐to‐date treatment algorithms. Despite biologics exhibiting the longest drug survival, IL‐17 and IL‐23 inhibitors were largely reserved for subsequent LOTs, potentially reflecting failed symptom control with non‐biologics. This may be due to the rarity of GPP, thus limiting empirical evidence for their efficacy, or may be due to their cost or burden on patients. In this study, oral drugs were commonly used as first‐line therapy, followed by biologics. Based on our experience, some patients achieve better control with oral drugs than currently available biologics. Our results demonstrated that not all patients treated with biologics had a drug survival exceeding 1 year and switching occurred with all biologics. The relatively short drug survival and frequent switching observed for biologics may be intrinsic to the recurrent and cyclic nature of GPP, reflecting the need to manage chronic and acute symptoms with different treatments. Investigating treatment patterns and drug survival within the context of GPP disease severity may help evaluate the efficacy of these treatments throughout the disease course. However, the variable treatment patterns also highlight that there are still considerable unmet needs for effective and well‐tolerated GPP treatments that optimally manage GPP throughout the disease course, enabling patients to remain on long‐term therapy where necessary. The identified treatment patterns and drug survival data in patients with GPP could facilitate the optimal management of patients with this disease. Future research could assess the impact of newly adopted treatments (such as bimekizumab, spesolimab, and deucravacitinib) and newly updated guidelines on treatment patterns and drug survival in patients with GPP.

## FUNDING INFORMATION

The study was supported and funded by Nippon Boehringer Ingelheim Co., Ltd.

## CONFLICT OF INTEREST STATEMENT

Yayoi Tada declares receiving research grants from AbbVie, Amgen, Boehringer Ingelheim, Bristol Myers Squibb, Eisai, Eli Lilly, Jimro, Kyowa Kirin, Leo Pharma, Maruho, Meiji Seika Pharma, Mitsubishi Tanabe, Sun Pharmaceutical Industries, Taiho Pharmaceutical, Torii Pharmaceutical, and UCB; honoria from AbbVie, Amgen, Boehringer Ingelheim, Bristol Myers Squibb, Eisai, Eli Lilly, Janssen, Jimro, Kyowa Kirin, Leo Pharma, Maruho, Mitsubishi Tanabe, Novartis, Sun Pharmaceutical Industries, Taiho Pharmaceutical, Torii Pharmaceutical, and UCB; and is an Editorial Board member of The Journal of Dermatology and a co‐author of this article. To minimize bias, they were excluded from all editorial decision‐making related to the acceptance of this article for publication. Jia Guan is an employee of Boehringer Ingelheim Pharmaceuticals Inc. Ryoko Iwasaki is an employee of Nippon Boehringer Ingelheim Co., Ltd. Akimichi Morita declares receiving research grants, consulting fees, and/or speaker's fees from AbbVie, Boehringer Ingelheim, Eisai, Eli Lilly, Janssen, Kyowa Kirin, Leo Pharma, Maruho, Mitsubishi Tanabe, Nichi‐Iko, Nippon Kayaku, Novartis, Sun Pharmaceutical Industries, Taiho Pharmaceutical, Torii Pharmaceutical, and Ushio.

## IRB APPROVAL STATUS

The study protocol was approved by the ethics committee of AMC Nishi‐Umeda Clinic (Osaka, Japan; AMC‐BI‐21‐010).

## Supporting information


Data S1.

## Data Availability

To ensure independent interpretation of clinical study results and enable authors to fulfill their role and obligations under the ICMJE criteria, Boehringer Ingelheim grants all external authors access to clinical study data pertinent to the development of the publication. In adherence with the Boehringer Ingelheim Policy on Transparency and Publication of Clinical Study Data, scientific and medical researchers can request access to clinical study data when it becomes available on Vivli – Center for Global Clinical Research Data, and earliest after publication of the primary manuscript in a peer‐reviewed journal, regulatory activities are complete, and other criteria are met. Please visit Medical & Clinical Trials | Clinical Research | MyStudyWindow for further information.
