# Supplementary material for: Treatment patterns and drug survival for generalized pustular psoriasis: A patient journey study using a Japanese claims database
Source: J Dermatol. 2024 Jan 12;51(3):391–402. doi: 10.1111/1346-8138.17097 (PMC11483928; doi:10.1111/1346-8138.17097)
Supplement: Supplementary file 1 — Data S1. [file JDE-51--s001.docx]

**Treatment patterns and drug survival for generalized pustular psoriasis: A patient journey study using a Japanese claims database**

Yayoi Tada, MD, PhD^1^, Jia Guan, MPH^2^, Ryoko Iwasaki, PhD^3^, Akimichi Morita, MD^4^

^1^Teikyo University Hospital, Tokyo, Japan; ^2^Boehringer Ingelheim Pharmaceuticals Inc., Ridgefield, CT, USA; ^3^Nippon Boehringer Ingelheim Co., Ltd., Tokyo, Japan; ^4^Nagoya City University Graduate School of Medical Sciences, Nagoya, Japan

**SUPPORTING INFORMATION**

**CONTENTS**

# Supplementary methods 4

## TABLE S1 List of treatments 4

## TABLE S2 Definitions used in the study 12

## ALGORITHMS FOR LINE OF THERAPY GENERATION 17

### Treatment patterns 17

### Drug survival 21

*Bio-switch 21*

# Supplementary RESULTS 23

## FIGURE S1 Treatment patterns for all general regimens among patients with GPP by line of therapy. 23

FIGURE S2 Frequency of (A) oral medications and (B) biologics at each line
of therapy. 24

## FIGURE S3 Combination patterns of GPP treatments: Combinations of (A) systemic medications and topical medications and (B) systemic medications and NSAIDs. 26

TABLE S3 Demographics and baseline characteristics of patients newly diagnosed with GPP between 2016 and 2021 28

TABLE S4 Time to next therapy (all specific regimens) 31

FIGURE S4 Treatment patterns for all general regimens among patients with GPP (A) with PsV and (B) without PsV. 32

FIGURE S5 Frequency of (A) oral medications and (B) biologics at each line
of therapy in patients with GPP with and without PsV. 34

TABLE S5 Drug survival of GPP treatments by general regimen in patients with GPP with and without PsV 36

FIGURE S6 Patterns in switching GPP treatment from one biologic to another biologic in patients with GPP (A) with PsV and (B) without PsV. 38

FIGURE S7 Treatment patterns for all general regimens in patients with GPP aged (A) ≥65 years and (B) ≥75 years. 40

FIGURE S8 Frequency of (A) oral medication and (B) biologics at each line
of therapy in patients with GPP aged ≥65 years and ≥75 years. 42

TABLE S6 Drug survival for patients with GPP aged ≥65 years, ≥75 years, and in the overall population 44

FIGURE S9 Frequency of switch to other biologics in patients with GPP
(A) aged ≥65 years and (B) ≥75 years. 46

**Supplementary methods**

**TABLE S1** List of treatments.

| **Treatment category 1** | **Treatment category 2** | **Treatment category 3** | **Main or supplementary** | **EphMRA ATC code, search term, or standard material code** | **Prescribed days** |
| --- | --- | --- | --- | --- | --- |
| Biologics | TNF-α inhibitors | Adalimumab | Main | L04B AND adalimumab | Dosing regimen: Q2W 14 days $\times$ unit For unit, use days  in “cnt” |
|  |  | Infliximab | Main | L04B AND infliximab | Dosing regimen: Q8W 56 days $\times$ unit |
|  |  | Certolizumab pegol | Main | L04B AND  certolizumab pegol | Dosing regimen: Q4W 28 days $\times$ unit |
|  |  | Etanercept | Main | L04B AND etanercept | Dosing regimen: Q1W 7 days $\times$ unit |
|  | IL-17 inhibitors | Secukinumab | Main | L04C AND secukinumab | Dosing regimen: Q4W 28 days $\times$ unit |
|  |  | Brodalumab | Main | L04C AND brodalumab | Dosing regimen: Q2W 14 days $\times$ unit |
|  |  | Ixekizumab | Main | L04C AND ixekizumab | Dosing regimen: Q4W 28 days $\times$ unit |
|  | IL-23 inhibitors | Risankizumab | Main | D05B AND risankizumab | Dosing regimen: Q12W 84 days $\times$ unit |
|  |  | Guselkumab | Main | D05B AND guselkumab | Dosing regimen: Q8W 56 days $\times$ unit |
|  |  | Ustekinumab | Main | L04C AND ustekinumab | Dosing regimen: Q12W 84 days $\times$ unit |
| Systemic corticosteroids | Systemic corticosteroids | Systemic corticosteroids | Main | H02A  AND betamethasone, dexamethasone, methylprednisolone, prednisolone, hydrocortisone, hydrocortisone sodium succinate, methylprednisolone sodium succinate | Use days in “cnt” |
| Systemic medication  (other than systemic corticosteroids) | Etretinate | Etretinate | Main | D05B AND etretinate | Use days in “cnt” |
|  | Cyclosporin | Cyclosporin | Main | L04X AND ciclosporin, cyclosporin | Use days in “cnt” |
|  | Apremilast | Apremilast | Main | D05B OR L04X  AND apremilast | Use days in “cnt” |
|  | Methotrexate | Methotrexate | Main | L01B OR L01D OR M01C AND methotrexate | Use days in “cnt” |
|  | Supplementary drug | Other oral medication | Supplementary | Immunosuppressant L04X AND tacrolimus with or without “hydrate” OR J04B AND diaphenylsulfone/dapsone OR J01A AND minocycline with or without “hydrochloride” OR L04X AND azathioprine | Use days in “cnt” |
|  |  |  | Supplementary | Antihistamines for  systemic use R06 | Use days in “cnt” |
| Topical therapy | Supplementary drug | Topical therapy | Supplementary | Topical vitamin D3 D05A  AND tacalcitol hydrate, calcipotriol, maxacalcitol, combination of calcipotriol hydrate, and betamethasone dipropionate, combination of maxacalcitol and betamethasone butyrate propionate | 28 days per prescription  For prescription, use “cnt” |
|  |  |  |  | Topical corticosteroids D07A AND Strongest: clobetasol propionate, diflorasone diacetate Very strong: betamethasone butyrate propionate, difluprednate, diflucortolone valerate, betamethasone dipropionate, fluocinonide, hydrocortisone butyrate propionate, mometasone furoate Strong: dexamethasone valerate, dexamethasone propionate, betamethasone valerate, fluocinolone acetonide, deprodone propionate Medium: prednisolone valerate acetate, clobetasone butyrate, triamcinolone acetonide, hydrocortisone butyrate, alclometasone dipropionate Weak: prednisolone, hydrocortisone | 28 days per prescription |
|  |  |  |  | Other topical medications D05X AND tacrolimus with or without “hydrate” AND “ointment” in product name  OR D05A AND salicylic acid | 28 days per prescription |
|  |  |  |  | Phototherapy Narrow-band UVB, PUVA Procedure code J054 Exclude skin laser irradiation therapy | Regimen: one procedure per week 7 days $\times$ procedure |
| Apheresis/plasma exchange | Apheresis/plasma exchange | Apheresis/plasma exchange | Main | GMA Procedure code J041-00 (three codes) | Regimen: one procedure per week 7 days $\times$ procedure |
|  |  |  |  | Plasma exchange Procedure code J039-00  (one code) | Regimen: One procedure per week 7 days $\times$ procedure |
| Arthritis treatment | Supplementary drug | Arthritis treatment | Supplementary | Tocilizumab M01C OR L04C AND tocilizumab | Regimen: Q4W 28 days $\times$ unit |
|  |  |  | Supplementary | NSAIDs  M01A | Use days in “cnt” |

Abbreviations: ATC, Anatomical Therapeutic Chemical EphMRA, European Pharmaceutical Market Research Association; GMA, granulocyte–monocyte apheresis; IL, interleukin; NSAID, non-steroidal anti-inflammatory drug; PUVA, psoralen plus ultraviolet A; QxW, every x weeks (where x represents the number of weeks); TNF-α, tumor necrosis factor alpha; UVB, ultraviolet B.

**TABLE S2** Definitions used in the study.

| **Baseline characteristics, covariates** | **ICD-10 code(s)** |
| --- | --- |
| With pregnancy | Z32.x, Z33, Z34.x, Z35.x |
| With other psoriasis | L40.0, L40.4, L40.8, L40.9 |
| With psoriatic arthritis | L40.5 |
| With cancer | C00-D48 |
| With fever | R50.8, R50.9 |
| With pain | R52 |
| With edema | R60 |

| **Comorbidities used for  CCI score calculation** | **ICD-10 codes** |
| --- | --- |
| Myocardial infarction | I21.x, I22.x, I25.2 |
| Congestive heart failure | I09.9, I11.0, I13.0, I13.2, I25.5, I42.0, I42.5–I42.9, I43.x, I50.x, P29.0 |
| Peripheral vascular disease | I70.x, I71.x, I73.1, I73.8, I73.9, I77.1, I79.0, I79.2, K55.1, K55.8, K55.9, Z95.8, Z95.9 |
| Cerebrovascular disease | G45.x, G46.x, H34.0, I60.x–I69.x |
| Dementia | F00.x–F03.x, F05.1, G30.x, G31.1 |
| Chronic pulmonary disease | I27.8, I27.9, J40.x–J47.x, J60.x–J67.x, J68.4, J70.1, J70.3 |
| Rheumatologic disease | M05.x, M06.x, M31.5, M32.x–M34.x, M35.1, M35.3, M36.0 |
| Peptic ulcer disease | K25.x–K28.x |
| Mild liver disease | B18.x, K70.0–K70.3, K70.9, K71.3–K71.5, K71.7, K73.x, K74.x, K76.0, K76.2–K76.4, K76.8, K76.9, Z94.4 |
| Diabetes without chronic complications | E10.0, E10.1, E10.6, E10.8, E10.9,E11.0, E11.1, E11.6, E11.8, E11.9,E12.0, E12.1, E12.6, E12.8, E12.9, E13.0, E13.1, E13.6, E13.8, E13.9,E14.0, E14.1, E14.6, E14.8, E14.9 |
| Diabetes with chronic complications | E10.2–E10.5, E10.7, E11.2–E11.5,E11.7, E12.2–E12.5, E12.7, E13.2–E13.5, E13.7, E14.2–E14.5, E14.7 |
| Hemiplegia or paraplegia | G04.1, G11.4, G80.1, G80.2, G81.x, G82.x,  G83.0–G83.4, G83.9 |
| Renal disease | I12.0, I13.1, N03.2–N03.7, N05.2–N05.7, N18.x, N19.x, N25.0, Z49.0–Z49.2, Z94.0, Z99.2 |
| Any malignancy, including lymphoma and leukemia, except malignant neoplasm of skin | C00.x–C26.x, C30.x–C34.x, C37.x–C41.x, C43.x,  C45.x–C58.x, C60.x–C76.x, C81.x–C85.x, C88.x,  C90.x–C97.x |
| Moderate or severe liver disease | I85.0, I85.9, I86.4, I98.2, K70.4, K71.1, K72.1, K72.9, K76.5, K76.6, K76.7 |
| Metastatic solid tumor | C77.x–C80.x |
| AIDS/HIV | B20.x–B22.x, B24.x |

| **GPP comorbidities of interest** | **ICD-10 code(s)** |
| --- | --- |
| Allergies |  |
| Allergic rhino conjunctivitis | J30.4 |
| Allergic contact dermatitis | L23.x |
| Autoimmune conditions |  |
| Psoriatic arthritis | L40.5 |
| Other psoriasis | L40.2, L40.4, L40.8, L40.9 |
| Bone |  |
| Osteoporosis | M80.0, M80.8, M81.0, M81.6, M81.8 |
| Cardiovascular conditions |  |
| Myocardial infarction | I21.x, I22.x |
| Stroke | I60.x, I61.x, I62.x, I63.x |
| Gastrointestinal conditions |  |
| Celiac disease | K90.0 |
| Crohn’s disease | K50.x |
| Diverticulitis | K57.x |
| Peptic ulcer disease | K25.x, K26.x, K27.x, K28.x |
| Ulcerative colitis | K51.x |
| Eye conditions |  |
| Uveitis | H20.x, H21.x |
| Infections |  |
| Tonsillitis | J03.x |
| Sinusitis | J32.x |
| Sepsis | A40.x, A41.x |
| Hormonal/metabolic conditions |  |
| Diabetes mellitus (type II) | E11.x  AND  At least one anti-diabetic treatment (insulin included) to occur within 30 days before or after the diagnosis code; the ATC codes for anti-diabetic treatments are the ones starting with A10X except A10D |
| Hyperlipidemia | E78.x  AND  At least one hyperlipidemia treatment to occur within 30 days before or after the diagnosis code; the ATC codes for hyperlipidemia treatments are the ones starting with C10A, AC10B, and C10C |
| Metabolic syndrome | E88.9 |
| Obesity | E66.x  OR  BMI ≥25 kg/m^2^ |
| Pulmonary conditions |  |
| Asthma | J45.x |
| Chronic obstructive pulmonary disease | J44.9 |
| Acute respiratory  distress syndrome | J80 |
| Others |  |
| Disseminated intravascular coagulation | D65 |
| Neutrophilic cholangitis | K83.0 |

| **Symptoms** | **ICD-10 code(s)** |
| --- | --- |
| Pain | R52 |
| Fatigue | R53 |
| Edema | R60 |
| Fever | R50.8, R50.9 |

Abbreviations: AIDS, autoimmune deficiency syndrome; ATC, Anatomical Therapeutic Chemical; BMI, body mass index; CCI, Charlson Comorbidity Index; GPP, generalized pustular psoriasis; HIV, human immunodeficiency virus;
ICD-10, International Classification of Diseases, 10^th^ Revision.ALGORITHMS FOR LINE OF THERAPY GENERATION

*Treatment patterns*

The algorithm to determine line of therapy (LOT) was based on the steps below.

*Step 1. Identify start and end date of each prescription of each medication.* For each medication listed in treatment category 3 of ANNEX 1 in the statistical and epidemiological analysis plan, we identified the start date according to the “actdate”. If there was more than one prescription of the same medication on the same day, we summed the “cnt” and calculated the prescribed days. When the same medication was prescribed before the end date of the previous prescription, we added the overlapped days to extend the end date of prescription. For drugs categorized in the same category of the category 3 level, but listed separately in “search terms”, we obtained the prescribed days for each drug according to the rules (i.e., first for betamethasone, and separately for dexamethasone, etc.). In episode calculation, the drugs under category 3 were merged, and the maximum prescribed days, not the sum of the overlap, were used to calculate gaps. Thus, we identified the end date.

*Step 2. Switch algorithms.* Based on all medications and sorted by the “actdate” and medications, a time gap over 28 days of a subsequent different medication was initiated, and no overlap observed between the two medications was considered as start of the subsequent LOT (e.g., if DIF(startdate) > 28 and (startdate – LAG(enddate)) > 0 and drugname ^= LAG(drugname) then LOTmark = 1); or if a time gap of no therapy over 3 months of a same sequential medication was considered as start of the subsequent LOT (e.g., if (startdate – LAG(enddate)) > 91 and drugname = LAG(drugname) then LOTmark = 1). Thus, the start date of LOT was defined as the date of LOTmark as 1, and the end date of LOT was defined as the start date of sequential LOT –1. If the LOT was the last one, then the end date of LOT was the end date of the last medication of that patient and marked as censor. According to the start date and end date of LOT in this step, the start date and end date of each medication in each LOT was updated.

A

B

1^st^ regimen: A

2^nd^ regimen: B

>28 days

A

B

1^st^ regimen: A+B

<28 days

<91 days

*Step 3. Add-on algorithms.* In each LOT in step 2, we kept the first prescription of each medication. If a medication for generalized pustular psoriasis (GPP) treatment was initially prescribed over 28 days later than the start date of the previous medication, it was also defined as a new LOT, and the start date of the new LOT was defined as the administration date of the later medication. According to the start date and end dates of LOTs in this step, we updated the start date and end date of each medication in each LOT.

◄, initial date of an agent; ◆, subsequent prescription date;

——, GPP therapy; , interval of no therapy.

>28 days

<28 days

A

B

C

1^st^ regimen: A+B

2^nd^ regimen: A+B+C

*Step 4. Discontinuation algorithms.* In each LOT in step 3, for only combination therapy, a time gap of discontinuation of one medication >3 months (in that LOT, end date of the last medication – the last end date of one medication >91 days) was marked as a new LOT. However, if there were multiple new LOTs generated, then we kept the mark only if the new LOT duration was >3 months (e.g., if LEAD(startdate) – startdate <91 days then LOTmark = −1). The end date of LOT for the current combination regimen was defined as the end date of the discontinued medication. The start date of the new LOT for the continued medication(s) added an additional day. Thus, we generated the LOT for step 4 and corresponding start date and end date for LOT, and for each medication in each LOT.

>28 days

A

B

1^st^ regimen: A

2^nd^ regimen: A+B

<3 months

>28 days

A

1^st^ regimen: A

2^nd^ regimen: A+B

3^rd^ regimen: A

1^st^ regimen: A

2^nd^ regimen: A+B

3^rd^ regimen: A

>3 months

B

◄, initial date of an agent; ◆, subsequent prescription date;

——, GPP therapy; , interval of no therapy.

*Step 5. Combine the same sequential regimens in one LOT.* If the consecutive regimens were the same, then they were regarded as in the same treatment line only if the time gap was <3 months (e.g., if regimen = LAG(regimen) and Lstartdate – LAG(Lenddate) < 91 then LOTmark = –1).

*Step 6. Generate LOT without supplementary drugs in the combination context.* If the supplementary drugs were identified as monotherapy in LOT in step 5, they were kept,
otherwise the supplementary drugs were deleted. We repeated steps 2–5.

*Step 7. Specific regimen with supplementary drug.* We added back the deleted supplementary drugs according to the start date and end date of the new LOT generated in step 6. If the treatment duration (sum of prescribed days) of the supplementary drugs was >3 months in that LOT, then we kept the supplementary drugs (otherwise, we deleted the supplementary drugs). Hence, we identified the corresponding specific regimen (category 3 drug name) and specific regimen (category 2 drug name).

*Step 8. Categorization to general regimen.* We identified the general regimen by grouping the specific regimen (category 2 drug name) by the prioritization order in ANNEX1.

*Drug survival*

Drug survival was determined by the number of prescribed days of each treatment, with prescribed days defined according to the treatment pattern definition in step 1 above.

Kaplan–Meier methods were used to calculate drug survival. When there were multiple episodes of one medication in a patient, the end date of the last episode was defined as “censored”, and the other episodes were defined as “events”; drug survival was then calculated from all episodes among users of that drug. Treatment duration over 1 or 2 years for each medication was generated by adding the sum of all episodes of that medication for each patient.

*Bio-switch*

After identifying the start and end dates of switching episodes in step 1 of the treatment patterns above, category 3 biologics were analyzed. Bio-switch was defined as a time gap over 28 days of a subsequent different medication initiated based on the definition in step 2. Switch frequency was calculated according to the last prescription date of each biologic, and was measured as the number of patients who switched to any other biologics (to other tumor necrosis factor alpha [TNF-α] inhibitors, other interleukin (IL)-17 inhibitors, and/or other IL-23 inhibitors). Regarding the percentage calculation, numerators were the numbers of patients who switched to other biologics and denominators were the number of biologics users.

**SUPPLEMENTARY RESULTS**

**FIGURE S1** Treatment patterns for all general regimens among patients with GPP by line of therapy.
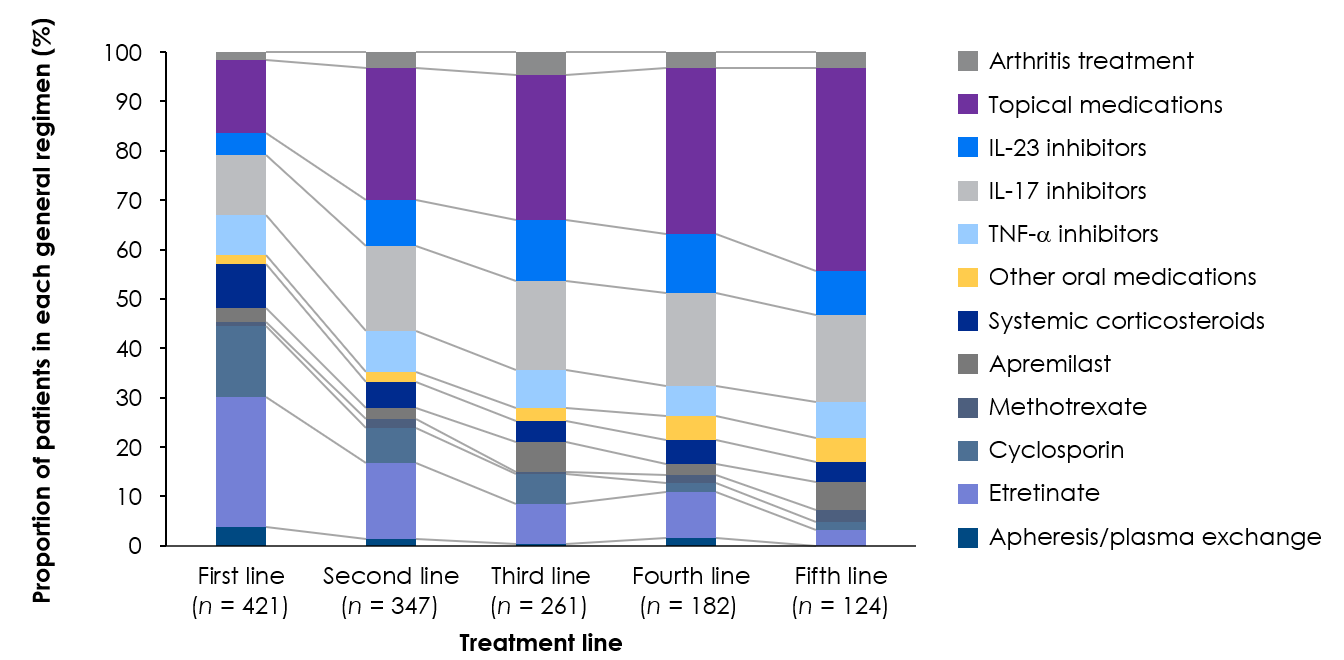


GPP, generalized pustular psoriasis; IL, interleukin; TNF-α, tumor necrosis factor alpha.

**FIGURE S2** Frequency of (A) oral medications and (B) biologics at each line
of therapy.

**A**


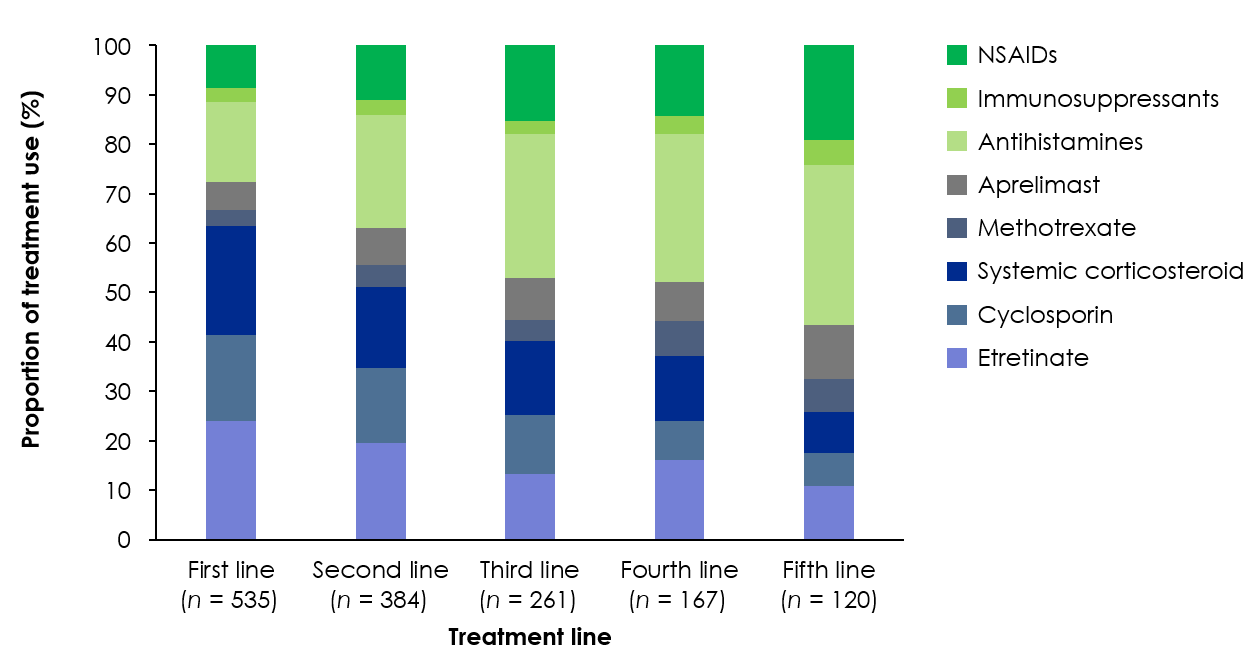


**B**
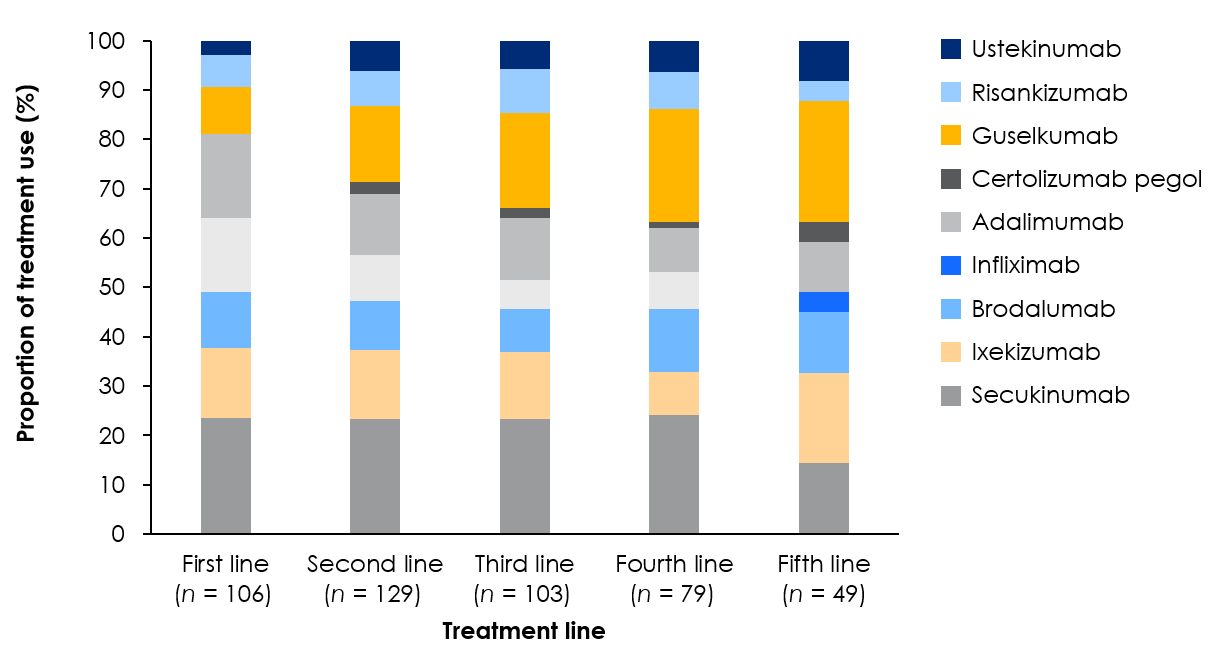


NSAID, non-steroidal anti-inflammatory drug.

**FIGURE S3** Combination patterns of GPP treatments: Combinations of (A) systemic medications and topical medications, and (B) systemic medications and NSAIDs.

**A**


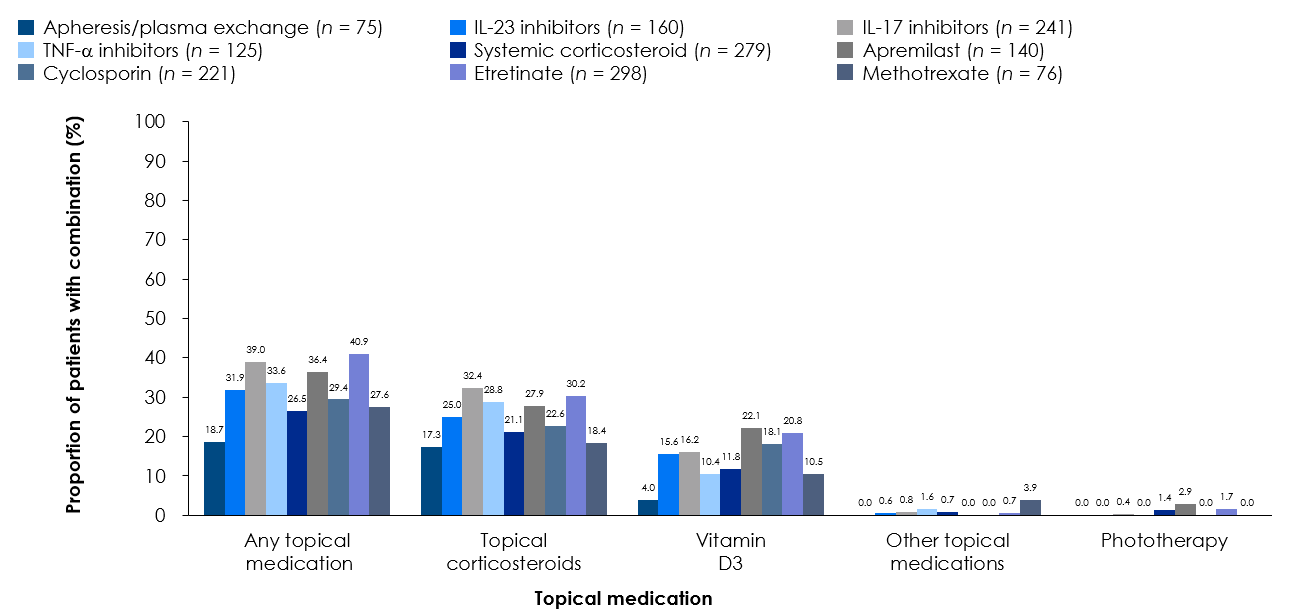


**B**


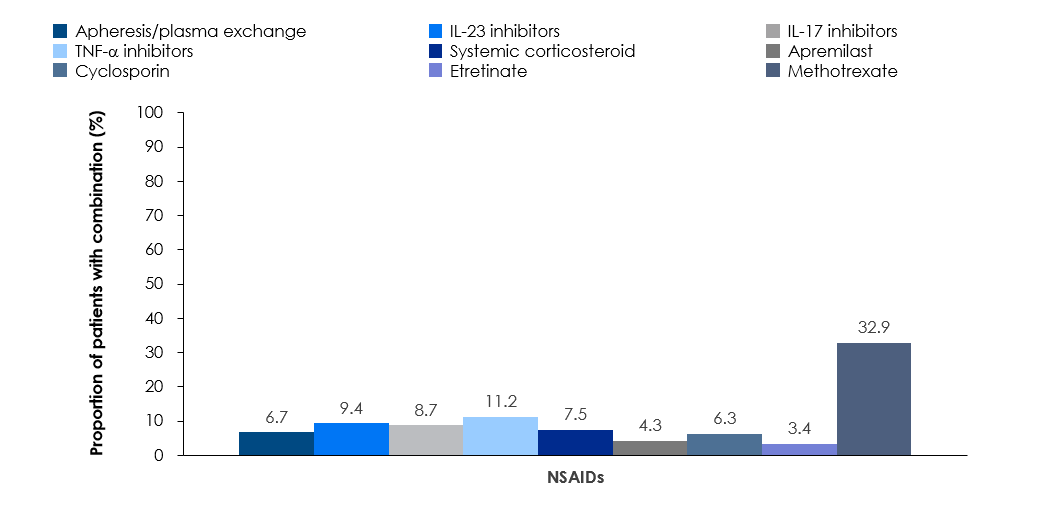


GPP, generalized pustular psoriasis; IL, interleukin; NSAID, non-steroidal anti-inflammatory drug; TNF-α, tumor necrosis factor alpha.

**TABLE S3** Demographics and baseline characteristics of patients newly diagnosed with GPP between January 1, 2016 and August 31, 2021.

| Demographics and baseline characteristics | Patients  (*N* = 434) |
| --- | --- |
| Age, mean (SD), years | 57.2 (19.9) |
| Age group, *n* (%), years |  |
| <15 | 15 (3.5) |
| 15–64 | 234 (53.9) |
| >64 | 185 (42.6) |
| Male, *n* (%) | 206 (47.5) |
| Total follow-up, median (IQR), days | 809 (570, 1206) |
| Number of beds in the hospital, *n* (%) |  |
| <200 | 13 (3.0) |
| 200–499 | 153 (35.3) |
| ≥500 | 268 (61.8) |
| Charlson Comorbidity Index Score, *n* (%) |  |
| 0 | 211 (48.6) |
| 1 | 92 (21.2) |
| 2 | 63 (14.5) |
| 3 | 25 (5.8) |
| ≥4 | 43 (9.9) |
| Selected comorbidities, *n* (%) |  |
| With psoriasis vulgaris | 159 (36.6) |
| With psoriatic arthritis | 41 (9.4) |
| With cancer | 53 (12.2) |
| With pregnancy | 2 (0.5) |
| Top five common comorbidities^†^, *n* (%) |  |
| Psoriasis vulgaris | 159 (36.6) |
| Hypertension | 79 (18.2) |
| Low back pain | 76 (17.5) |
| Eczema | 73 (16.8) |
| Constipation | 63 (14.5) |
| Top five concomitant medications, *n* (%) |  |
| Lidocaine hydrochloride/adrenaline | 164 (37.8) |
| Isotonic sodium chloride solution | 140 (32.3) |
| Acetaminophen | 110 (25.4) |
| White petrolatum | 99 (22.8) |
| Heparinoid | 90 (20.7) |

**^†^**Identified by disease codes as follows: ICD-10 code L40.0 psoriasis vulgaris 6961004; standard disease code 8833421 hypertension; 8840829 low back pain; 8840042 constipation; 6923002 eczema. Standard disease code 6961004 psoriasis vulgaris was *N* = 128 (29.5%).

Abbreviations: GPP, generalized pustular psoriasis; ICD-10, International Classification of Diseases, 10^th^ Revision; IQR, interquartile range; SD, standard deviation.

**TABLE S4** Time to next therapy (all specific regimens).

| Time to next therapy | First line | All lines |
| --- | --- | --- |
| Total regimen numbers, *N* | 347 | 1137 |
| Median, months | 2.8 | 3.3 |
| IQR | 5.2 | 5.4 |
| Min, max | 0.03–48.07 | 0.03–52.97 |

Abbreviations: IQR, interquartile range; max, maximum; min, minimum.

**FIGURE S4** Treatment patterns for all general regimens among patients with newly diagnosed GPP (A) with PsV and (B) without PsV.

**A**
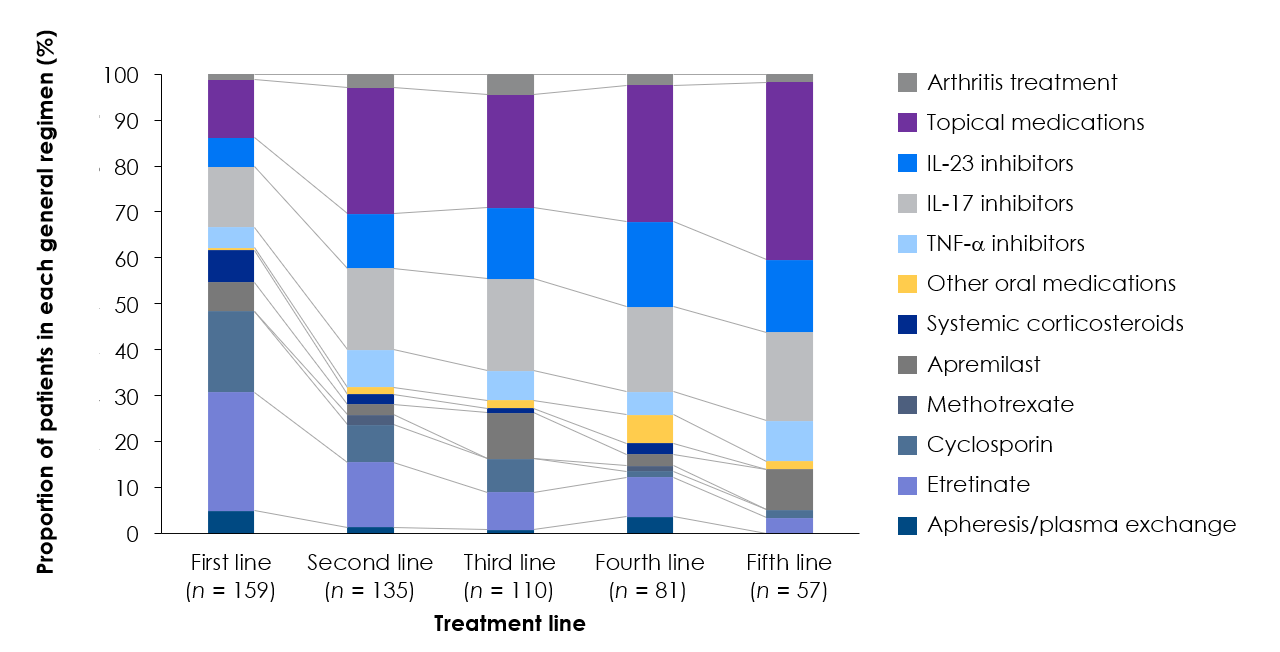
**B**
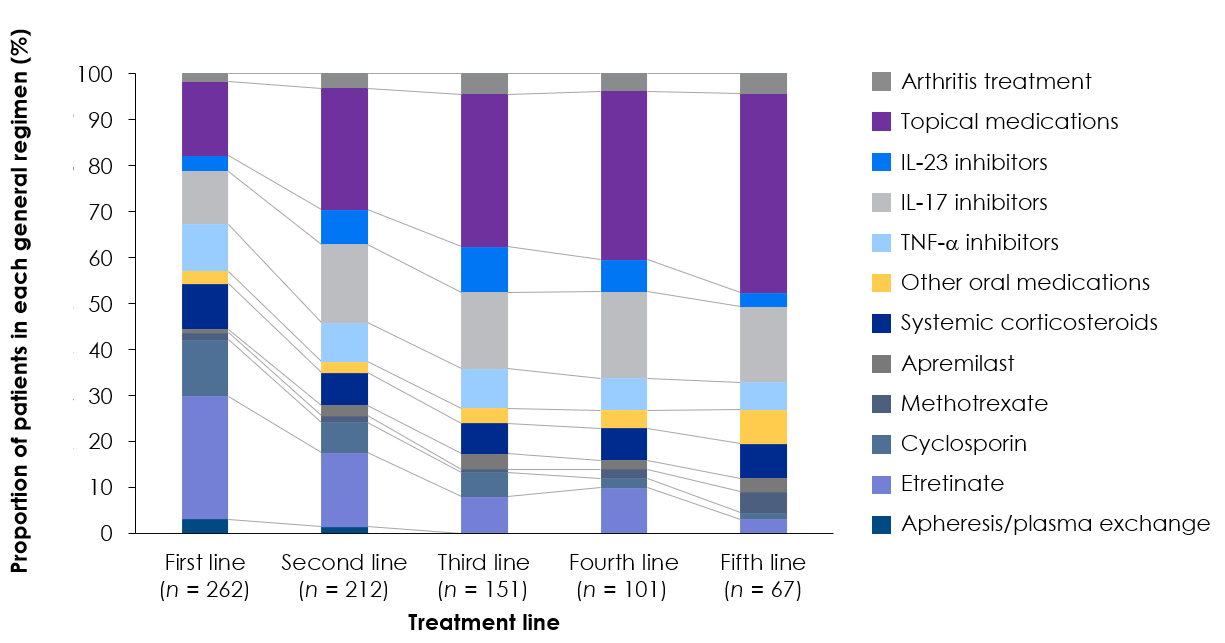


GPP, generalized pustular psoriasis; IL, interleukin; PsV, psoriasis vulgaris;
TNF-α, tumor necrosis factor alpha.

**FIGURE S5** Frequency of (A) oral medications and (B) biologics at each line of therapy in patients with newly diagnosed GPP, with and without PsV.

**A**


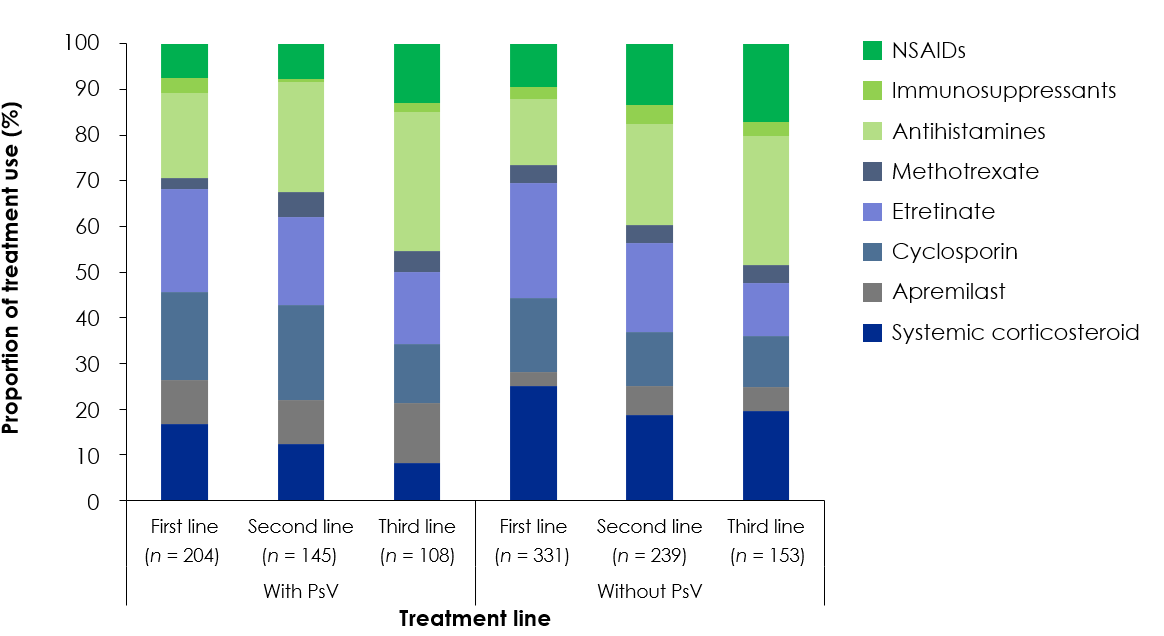


**B**


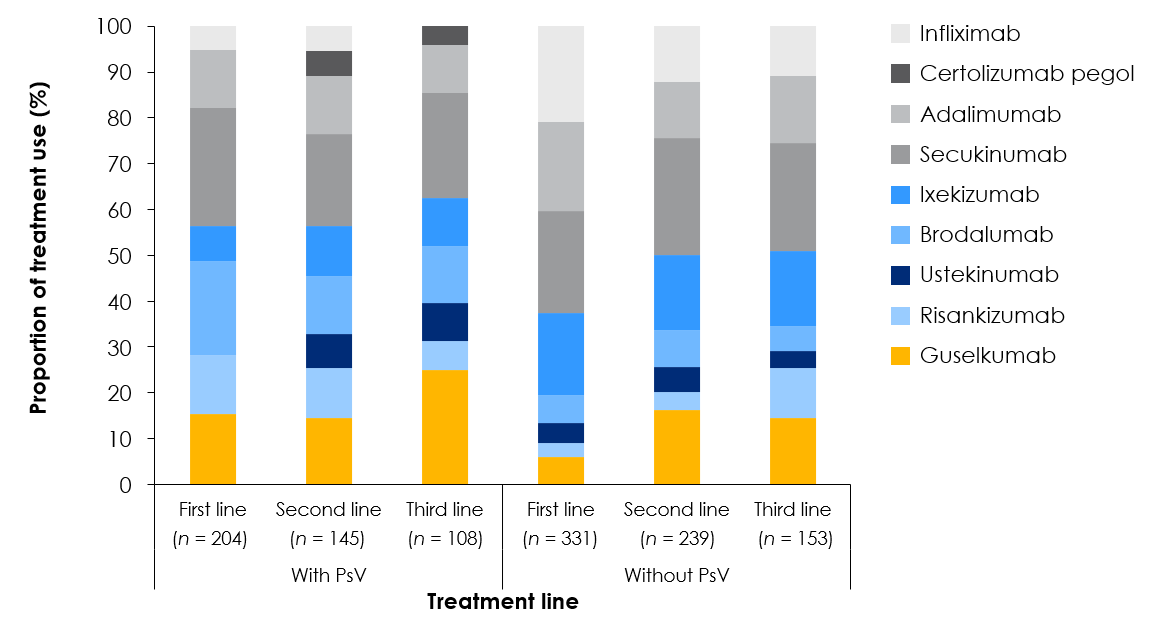


GPP, generalized pustular psoriasis; NSAID, non-steroidal anti-inflammatory drug;
PsV, psoriasis vulgaris.

**TABLE S5** Drug survival of GPP treatments by general regimen in patients with GPP with and without PsV.

| **Drug category and name** | | **Cohort with PsV** | | **Cohort without PsV** | | **Overall population** | |
| --- | --- | --- | --- | --- | --- | --- | --- |
|  |  | **Number of treatment episodes** | **Median drug survival, months  (95% CI)** | **Number of treatment episodes** | **Median drug survival, months  (95% CI)** | **Number of treatment episodes** | **Median drug survival, months  (95% CI)** |
| Oral medication | Apremilast | 53 | 8.6 (2.8–15.2) | 34 | 10.1 (3.9–18.6) | 87 | 10.1 (3.9–15.0) |
|  | Cyclosporin | 63 | 5.4 (3.1–9.6) | 76 | 5.7 (2.8–9.5) | 139 | 5.6 (4.0–7.9) |
|  | Etretinate | 72 | 13.1 (6.0–18.5) | 127 | 6.8 (4.7–9.7) | 199 | 8.8 (5.8–11.8) |
|  | Methotrexate | 27 | 4.9 (4.1–13.5) | 64 | 12.4 (7.9–35.9) | 91 | 12.0 (4.9–19.3) |
|  | Systemic corticosteroid | 50 | 5.2 (2.1–10.9) | 142 | 2.8 (1.7–6.3) | 192 | 4.3 (2.2–6.9) |
| Other oral medication | Antihistamines | 168 | 9.7 (6.9–12.7) | 258 | 6.2 (4.2–10.0) | 426 | 8.2 (5.6–10.1) |
|  | Other immune suppressants | 24 | 0.7 (0.4–15.1) | 55 | 0.8 (0.5–3.7) | 79 | 0.8 (0.5–3.7) |
| TNF-α inhibitors | Adalimumab | 22 | 9.6 (3.3–31.8) | 25 | 19.8 (11.2–22.4) | 47 | 17.8 (6.5–22.4) |
|  | Certolizumab pegol | 5 | 8.4 (0.9–13.9) | 1 | 4.7 (NC–NC) | 6 | 8.4 (4.7–12.1) |
|  | Infliximab | 4 | 31.8 (7.5–38.5) | 19 | 16.8 (9.3–24.9) | 23 | 22.1 (9.3–26.1) |
| IL-17 inhibitors | Brodalumab | 20 | 11.4 (6.5–12.8) | 14 | 25.8 (4.1–42.7) | 34 | 12.6 (8.2–20.8) |
|  | Ixekizumab | 17 | 17.7 (5.4–24.8) | 28 | 15.5 (13.7–24.3) | 45 | 15.5 (10.7–23.8) |
|  | Secukinumab | 28 | 16.1 (14.0–21.0) | 38 | 22.4 (16.8–36.4) | 66 | 19.6 (16.1–26.7) |
| IL-23 inhibitors | Guselkumab | 33 | 11.2 (7.5–19.8) | 22 | 14.9 (9.3–25.1) | 55 | 14.0 (9.3–19.8) |
|  | Risankizumab | 14 | 10.9 (7.0–15.0) | 12 | 16.9 (11.2–21.0) | 26 | 13.5 (10.1–15.6) |
|  | Ustekinumab | 6 | 11.2 (11.2–16.8) | 8 | 18.2 (15.0–53.2) | 14 | 16.8 (11.2–19.6) |

Abbreviations: CI, confidence interval; GPP, generalized pustular psoriasis; IL, interleukin;
NC, non-calculable; PsV, psoriasis vulgaris; TNF-α, tumor necrosis factor alpha*.*

**FIGURE S6** Patterns in switching GPP treatment from one biologic to another in patients with newly diagnosed GPP (A) with PsV or (B) without PsV.

**A

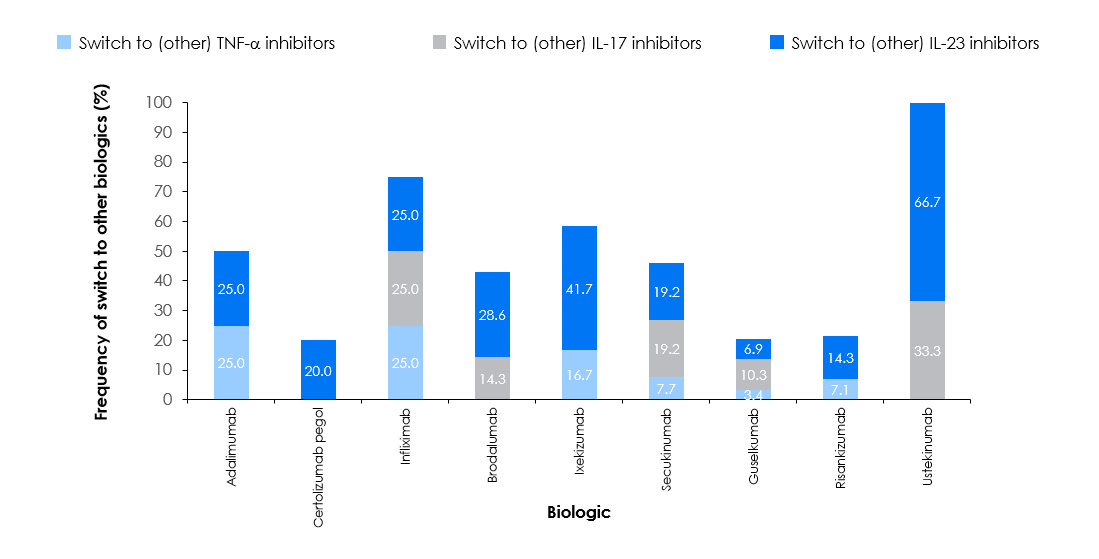
**

**B**


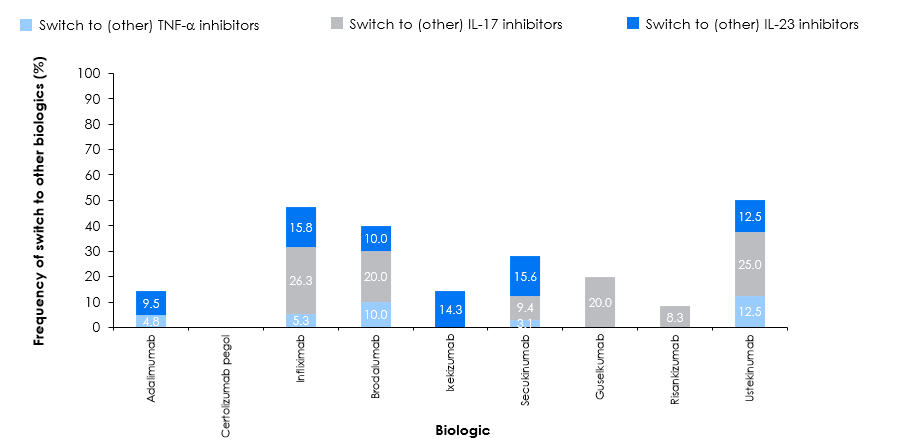

GPP, generalized pustular psoriasis; IL, interleukin; PsV, psoriasis vulgaris; TNF-α, tumor necrosis factor alpha

**FIGURE S7** Treatment patterns for all general regimens in patients with newly diagnosed GPP aged (A) ≥65 years and (B) ≥75 years.

**A**


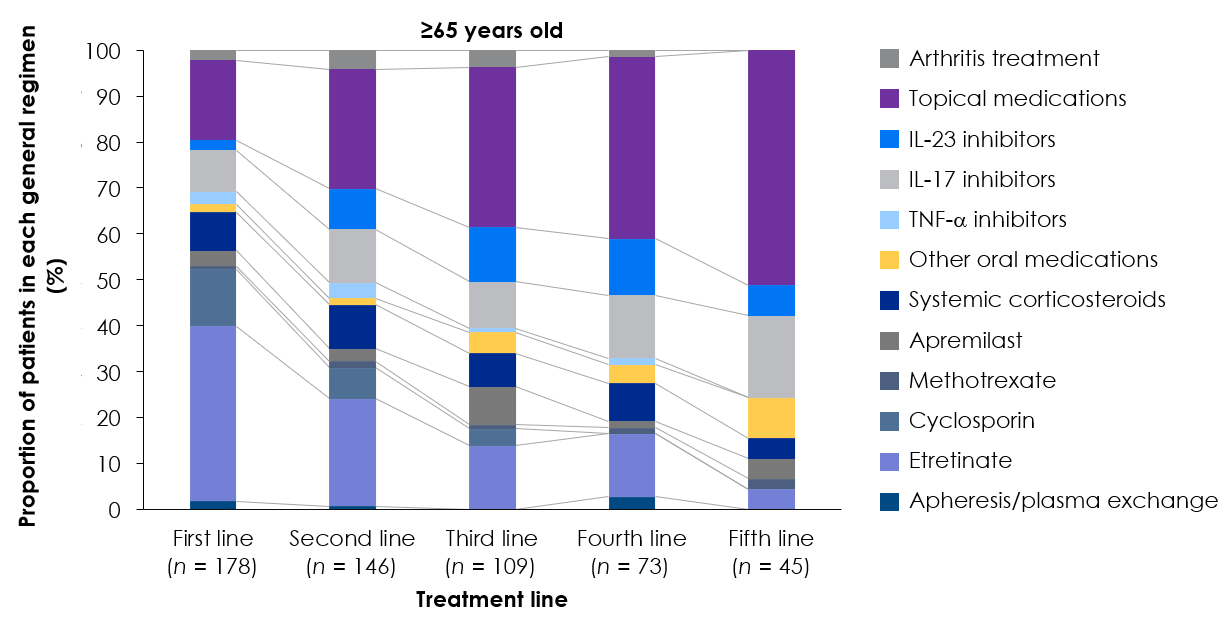


**B**


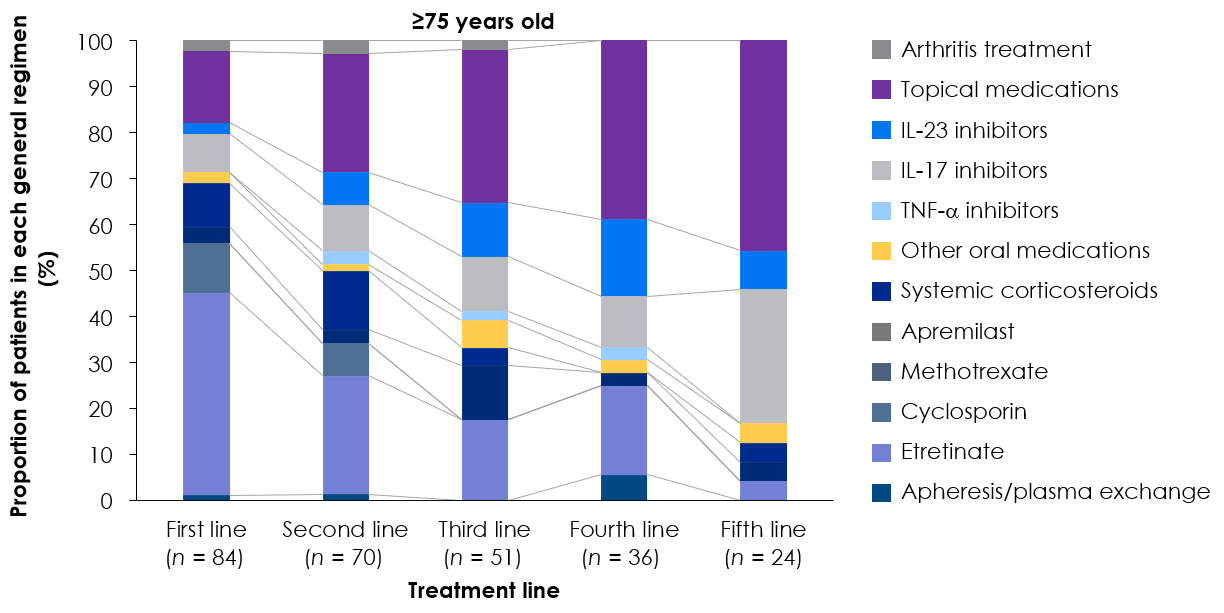


GPP, generalized pustular psoriasis; IL, interleukin; TNF-α, tumor necrosis factor alpha.**FIGURE S8** Frequency of (A) oral medication and (B) biologics at each line of therapy in patients with GPP aged ≥65 years and ≥75 years.

**A**


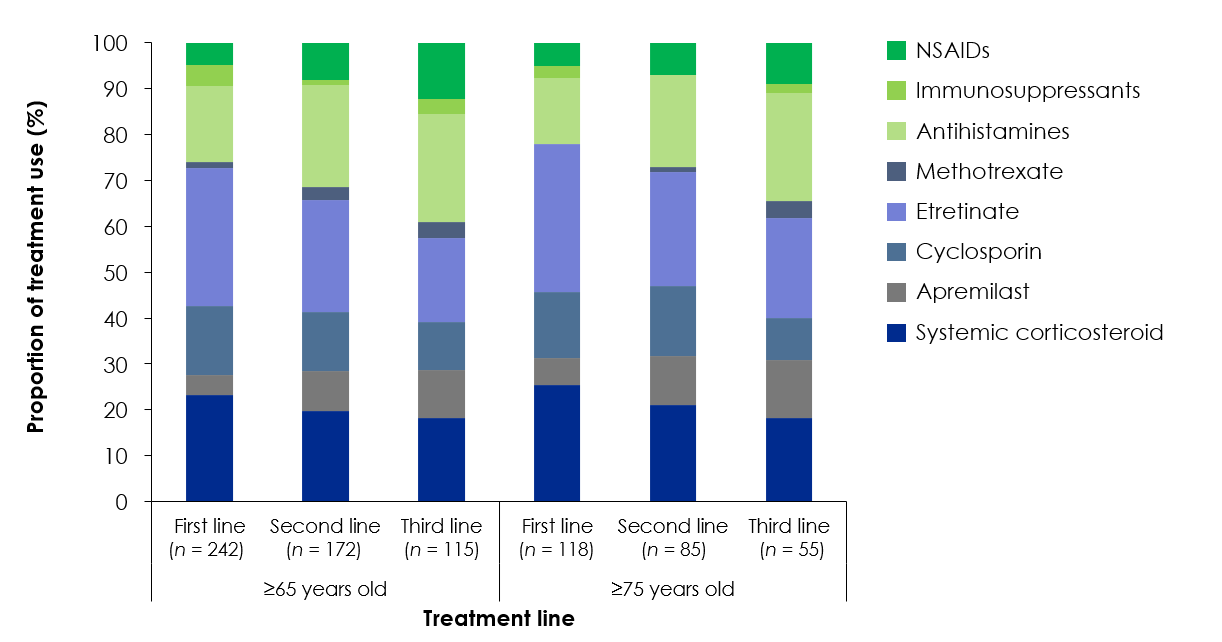


**B**


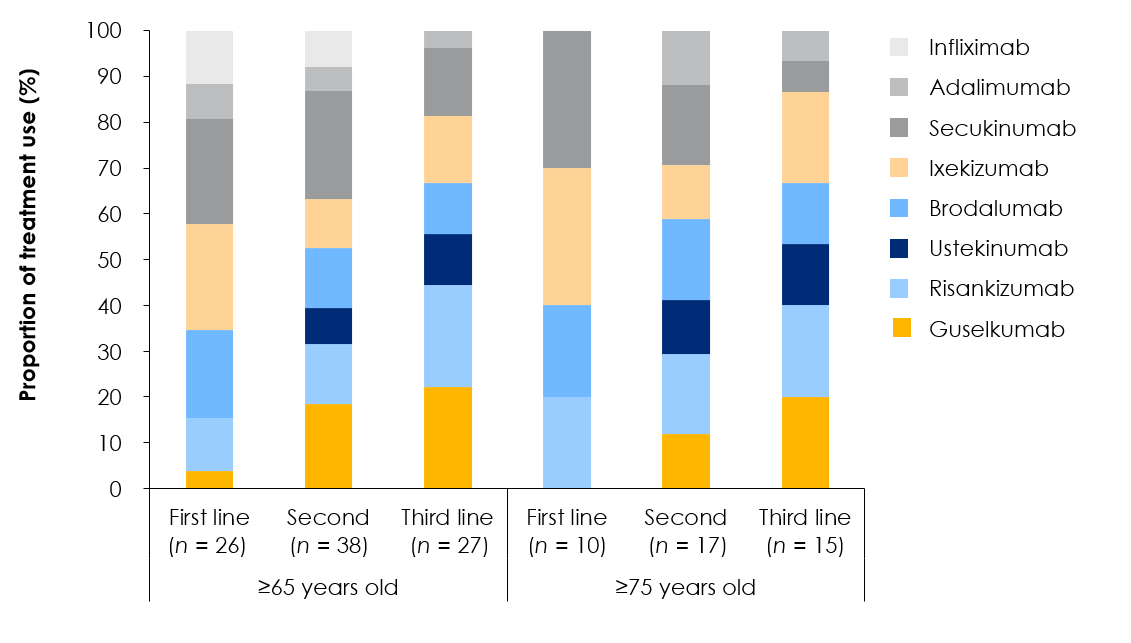


GPP, generalized pustular psoriasis; NSAID, non-steroidal anti-inflammatory drug.

**TABLE S6** Drug survival for patients with newly diagnosed GPP aged ≥65 years, ≥75 years, and in the overall population.

| **Drug category and name** | | **≥65-year-old cohort** | | **≥75-year-old cohort** | | **Overall population** | |
| --- | --- | --- | --- | --- | --- | --- | --- |
|  |  | **Number of treatment episodes** | **Median of drug survival months**  **(95% CI)** | **Number of treatment episodes** | **Median of drug survival months**  **(95% CI)** | **Number of treatment episodes** | **Median of drug survival months**  **(95% CI)** |
| Oral medication | Apremilast | 43 | 11.7 (2.8–16.1) | 22 | 11.7 (2.8–21.4) | 87 | 10.1 (3.9–15.0) |
|  | Cyclosporin | 51 | 7.3 (2.1–9.6) | 29 | 4.0 (1.4–12.5) | 139 | 5.6 (4.0–7.9) |
|  | Etretinate | 114 | 9.0 (5.4–14.0) | 58 | 7.7 (4.3–17.6) | 199 | 8.8 (5.8–11.8) |
|  | Methotrexate | 20 | 26.5 (4.9–26.5) | 7 | 4.9 (4.9–NC) | 91 | 12.0 (4.9–19.3) |
|  | Systemic corticosteroid | 96 | 2.3 (1.3–6.0) | 48 | 2.3 (1.3–8.0) | 192 | 4.3 (2.2–6.9) |
| Other oral medication | Antihistamines | 160 | 7.7 (4.0–10.2) | 71 | 7.7 (4.1–14.0) | 426 | 8.2 (5.6–10.1) |
|  | Other immune suppressants | 38 | 0.5 (0.3–1.4) | 9 | 0.5 (0.4–5.6) | 79 | 0.8 (0.5–3.7) |
| TNF-α inhibitors | Adalimumab | 7 | 2.6 (1.0–22.4) | 4 | 12.5 (1.0–22.4) | 47 | 17.8 (6.5–22.4) |
|  | Certolizumab pegol | 0 | 0 | 0 | 0 | 6 | 8.4 (4.7–12.1) |
|  | Infliximab | 5 | 16.8 (4.3–45.4) | 0 | 0 | 23 | 22.1 (9.3–26.1) |
| IL-17 inhibitors | Brodalumab | 11 | 12.1 (4.1–27.0) | 6 | 17.0 (4.9–42.7) | 34 | 12.6 (8.2–20.8) |
|  | Ixekizumab | 16 | 14.3 (4.6–18.4) | 10 | 18.4 (4.7–24.8) | 45 | 15.5 (10.7–23.8) |
|  | Secukinumab | 19 | 17.4 (4.7–37.4) | 6 | 19.3 (3.7–37.4) | 66 | 19.6 (16.1–26.7) |
| IL-23 inhibitors | Guselkumab | 15 | 13.1 (7.5–25.1) | 6 | 11.1 (7.5–25.1) | 55 | 14.0 (9.3–19.8) |
|  | Risankizumab | 11 | 11.2 (5.6–15.9) | 5 | 10.5 (5.6–11.2) | 26 | 13.5 (10.1–15.6) |
|  | Ustekinumab | 4 | 15.5 (11.2–19.6) | 2 | 15.4 (11.2–19.6) | 14 | 16.8 (11.2–19.6) |

Abbreviations: CI, confidence interval; GPP, generalized pustular psoriasis; IL, interleukin;
NC, non-calculable; TNF-α, tumor necrosis factor alpha.

**FIGURE S9** Frequency of switch to other biologics in patients with newly diagnosed GPP aged (A) ≥65 years and (B) ≥75 years.

**A**


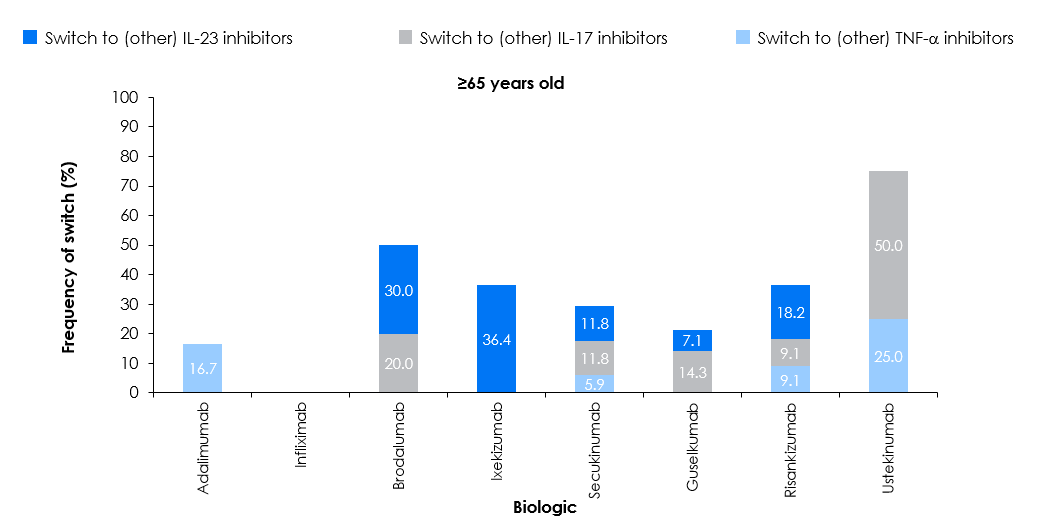


**B**


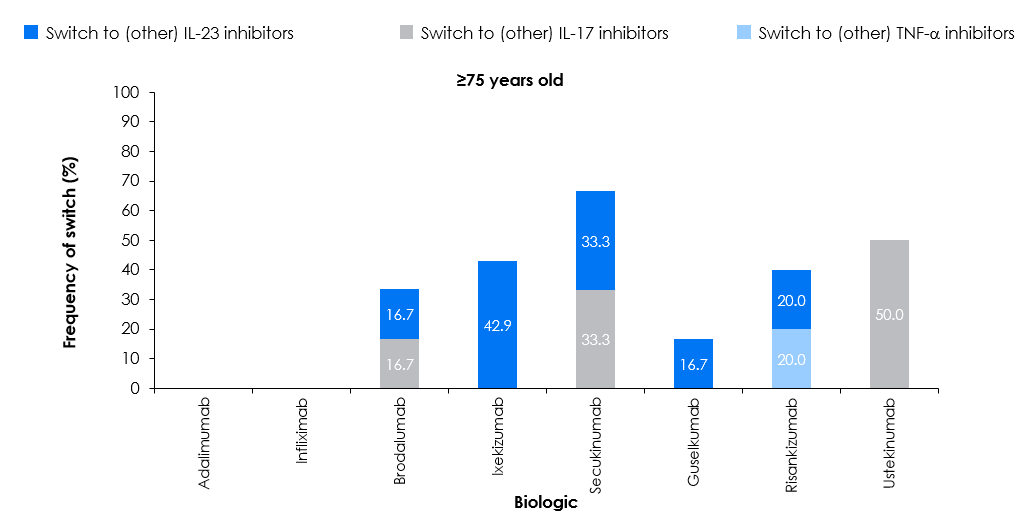


GPP, generalized pustular psoriasis; IL, interleukin; TNF-α, tumor necrosis factor alpha.
